# Discovery of a novel envelope protein derived from simian retrovirus 2 for pseudotyping retroviral vectors used for production of CAR immune cells

**DOI:** 10.1038/s41467-026-72024-4

**Published:** 2026-04-23

**Authors:** Moonjung Jeun, Yeongrin Kim, Heung Kyoung Lee, Ji U Choi, Hye Gwang Jeong, Chi Hoon Park

**Affiliations:** 1 Bio & Drug Discovery Division, Korea Research Institute of Chemical Technology, Daejeon, Republic of Korea; 2 College of Pharmacy, Chungnam National University, Daejeon, Republic of Korea; 3 Medicinal Chemistry and Pharmacology, University of Science and Technology, Daejeon, Republic of Korea

**Keywords:** Gene therapy, Genetic transduction

## Abstract

In this study, we develop a simian retrovirus 2 pseudotyped retrovirus (SRV2 RV) for the generation of CAR-based immune cells. The SRV2 RV exhibits superior gene transduction efficiency in both T cells and NK cells compared to feline endogenous retrovirus (RD114) pseudotyped retrovirus (RD114 RV) or vesicular stomatitis virus glycoprotein (VSV-G) pseudotyped lentivirus (VSV-G LV). Among the various SRV pseudotypes tested, only the SRV2 RV successfully transduces genes into immune cells. Unlike the SRV2 RV, however, lentivirus pseudotyped with the SRV2 envelope glycoprotein (ENV) fails to mediate gene transduction into T cells. CAR-T and NK cells generated using SRV2 RV demonstrate substantial anticancer activity both in vitro and in preclinical models. In conclusion, our findings highlight the SRV2 RV as a highly effective platform for producing CAR-based immune cells.

## Abstract (web summary)

It remains to be explored whether the SRV2 envelope can improve gene delivery to immune cells. The authors here show that SRV2 pseudotyped retroviral vectors enhance immune cell transduction over RD114 vectors and VSV-G lentiviral vectors, and efficiently generated CAR-T and CAR-NK cells in vitro.

## Introduction

Pseudotyped retrovirus and lentivirus are invaluable tools for gene transduction into target cells. They facilitate the integration of the transduced genes into target cell’s genome, allowing a long-term expression^1^. In manufacturing CAR-T cells, the production of high-quality viral vectors is essential for the success of therapy^2^. Several ENVs, including VSV-G, gibbon ape leukemia virus (GALV), and RD114, have been widely utilized for this purpose^3^.

SRV, classified as type-D retroviruses, was first identified from a mammary tumor in a rhesus monkey^4^. They exhibit a broad cell tropism, infecting both lymphoid and non-lymphoid cells of macaques, and can cause immunodeficiency syndromes in Asian macaques^5–7^. Simian type D retroviruses, including SRV1 through SRV8, demonstrate cross-interference with one another^8^. Furthermore, they interfere with type C retroviruses, such as RD114, baboon endogenous virus (BaEV), spleen necrosis virus (SNV) of chickens or ducks, and avian reticuloendotheliosis virus (REV)^9,10^. This interference occurs due to their shared use of the same entry receptor^11–13^. The receptor gene for RD114 and SRV is located on human chromosome 19, and has been identified through cDNA library studies as a neutral amino acid transporter, specifically ASCT2^14,15^.

Glycoproteins derived from RD114 have commonly been used to pseudotype retroviruses for gene transduction into immune cells to produce CAR-T or NK cells^16^. VSV-G LV is the most widely utilized for gene transduction into primary and diverse cell lines^17^. However, no studies to date have reported pseudotyping retroviruses or lentiviruses with SRV glycoproteins.

In this study, we demonstrate, for the first time, that pseudotyping retroviral vectors with SRV2 represents a potent strategy for gene transduction into immune cells. CAR-T and NK cells generated using SRV2 RV exhibit robust antitumor activity both in vitro and in vivo. These findings establish SRV2 RV as an effective platform for the production of CAR-engineered immune cells and highlight their potential to advance cell-based cancer immunotherapy.

## Results

### SRV2 pseudotyping enables stable and high-titer retrovirus production

**Fig. 1:**
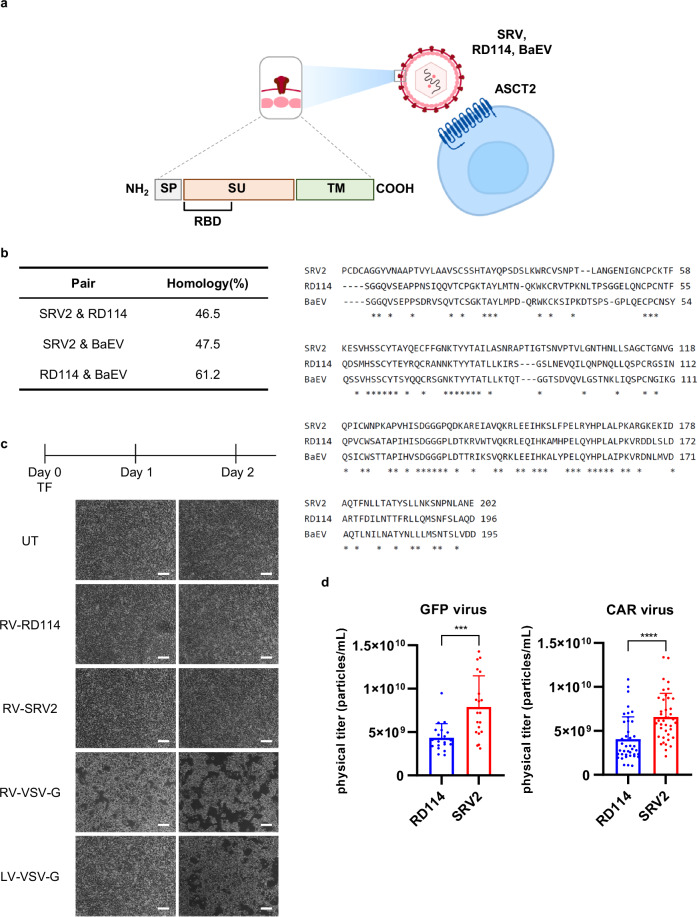
SRV2 ENV, which shares RBD similarity with RD114, supports high-titer and stable retrovirus production. **a** Schematic illustration of interaction between viral envelope and receptor enabling cellular entry. **b** (Right) Alignment of the receptor-binding domain (RBD) sequences from SRV2 ENV (AAA47563.1), RD114 ENV (AAA47563.1), and BaEV ENV (BAA00924.1). Conserved residues are indicated by asterisks. (Left) Pairwise sequence homology (%) across RBD region in ENVs. **c** Representative phase-contrast images of HEK293T cells producing retro- or lentiviruses pseudotyped with SRV2, RD114, or VSV-G (10× objective, scale bar, 10 µm; 50 ms exposure). TF denotes transfection, and UT denotes untrasfected cells. Images were collected at 1 and 2 days after transfection. **d** Physical titers of SRV2 RV and RD114 RV encoding *GFP* or CAR, quantified by qRT-PCR. Data are presented as mean ± s.d. (GFP virus, *n* = 20 biologically independent samples; CAR virus, *n* = 40 biologically independent samples). Statistical significance was assessed using two-tailed Mann–Whitney tests (***P = 0.0004 for GFP virus; ****P < *0.0001 for CAR virus*). Source data are provided as a Source Data file.

Viral ENVs recognize the receptors on surface of target cells and subsequently induce fusion between the viral and cellular membranes^18,19^. Various cellular receptors have been identified as entry points for retroviruses. They are sodium-dependent phosphate transporters including *SLC20A1* (PiT1) and *SLC20A2* (PiT2), the cationic amino acid transporter *SLC7A1* (MCAT), the glucose transporter *SLC2A1* (Glut-1), the riboflavin transporter *SLC5A1* (huPAR-2), and the glutamate/neutral amino acid transporters including *SLC1A4* (ASCT1) and *SLC1A5* (ASCT2)^20^. RD114 RV is the most commonly used for generating CAR-T cells. RD114 utilizes ASCT2, which is highly expressed in hematopoietic lineages, as an entry receptor. Similarly, SRV has been reported to use ASCT2 as an entry receptor (Fig. 1a)^14,20,21^. We found that the receptor-binding domain sequence of SRV2 ENV shows high homology with the corresponding domains of RD114 and BaEV ENVs (Fig. 1b). This led us to hypothesize that SRV2 RV could efficiently transduce genes into immune cells like RD114 RV. To test this hypothesis, we designed the experiment to compare the transduction efficiency of SRV2 RV with that of RD114 RV in T, NK and B cells. For this, we produced SRV2 or RD114 RV with 293 T cells. No damage to 293 T cells was observed during the production of the SRV2 RV, which ensures the stable production of SRV2 RV (Fig. 1c). Moreover, the viral titer of SRV2 RV was substantially higher than that of RD114 RV (Fig. 1d). This indicates that SRV2 pseudotyping may serve as an effective strategy for RV based gene transduction.

### SRV2 RV efficiently transduce genes into immune cells

**Fig. 2:**
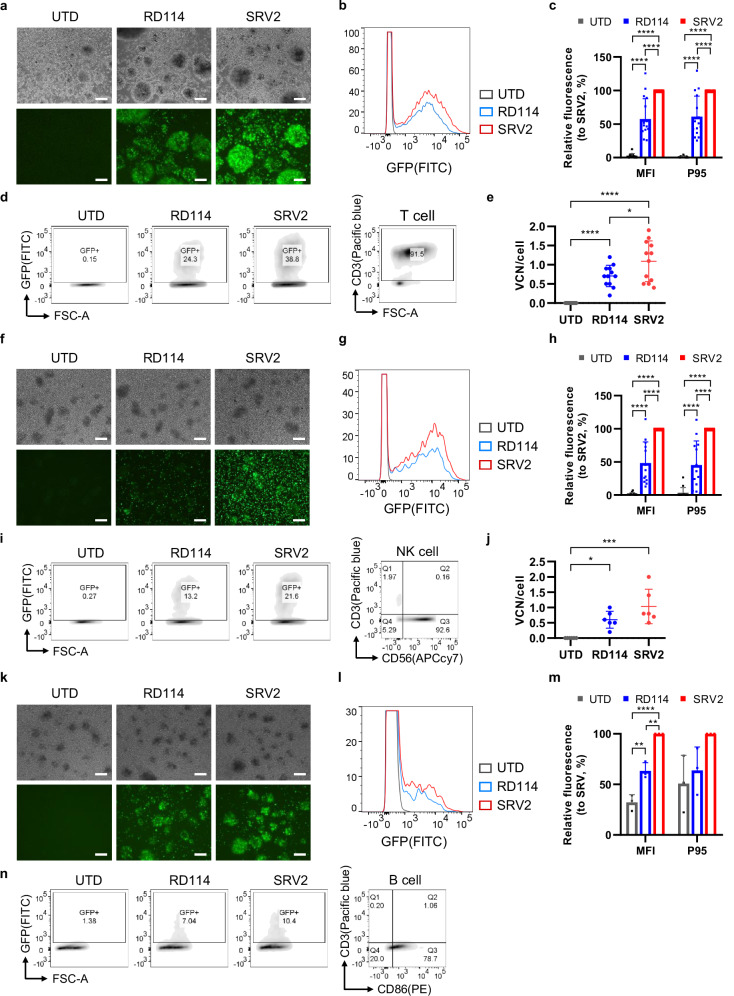
Enhanced gene delivery to primary human immune cells by SRV2 RV compared to RD114 RV. RD114 RV or SRV2 RV were used to transduce primary human T cells (**a**–**e**), NK cells (**f**–**j**), and B cells (**k**–**n**). Imaging, flow cytometric analyses and VCN analyses were performed 3–5 days post-transduction for T and B cells, and 6–8 days post-transduction for NK cells. **a**, **f**, **k** Representative bright-field and fluorescence images of transduced T, NK, and B cells (10× objective; scale bar, 10 µm; 50 ms exposure for bright-field and 100 ms exposure for fluorescence field). **b**, **d**, **g**, **i**, **l**, **n** Flow cytometry histograms and density plots showing *GFP* expression in T, NK, and B cells transduced with RD114 RV or SRV2 RV. Immune subsets were confirmed by lineage markers (T, CD3⁺; NK, CD3⁻CD56⁺; B, CD3⁻CD86⁺). Gating strategies are provided in Supplementary Fig. 6. **c**, **h**, **m** Quantification of *GFP* expression based on mean fluorescence intensity (MFI) and 95th percentile fluorescence (P95). Data are mean ± s.d. (T, *n* = 16 biologically independent replicates; NK, *n* = 13 biologically independent replicates; B, *n* = 3 biologically independent replicates). Statistical significance was determined by ordinary one-way ANOVA with Tukey’s post-hoc test. Exact *P* values for the indicated comparisons were as follows: in panel m, UTD vs RD114, *P* = 0.0026 and RD114 vs SRV2, *P* = 0.0011. **e**, **j** Vector copy number (VCN) in genomic DNA from *GFP* transduced T and NK cells. Data are mean ± s.d. (T, *n* = 12 biologically independent replicates; NK, *n* = 6 biologically independent replicates). Statistical analysis was performed using ordinary one-way ANOVA with Tukey’s post-hoc test. Exact *P* values for the indicated comparisons were as follows: in panel e, RD114 vs SRV2, *P* = 0.0272; in panel j, UTD vs RD114, *P* = 0.0072 and UTD vs SRV2, *P* = 0.0145. UTD, untransduced cells; RD114, cells transduced with RD114 RV; SRV2, cells transduced with SRV2 RV. *P < 0.05, **P < 0.01, ***P < 0.001, ****P < 0.0001. Across all panels, gray indicates UTD, blue indicates RD114, and red indicates SRV2. Source data are provided as a Source Data file.

Having demonstrated that SRV2 RV can be stably produced at high titers, we next examined whether it could effectively mediate gene transduction into immune cells. The efficiency of gene transduction into T, NK, and B cells was assessed based on fluorescence intensity (Fig. 2). SRV2 RV showed substantially higher transduction efficiency than RD114 RV in all three immune cell types (Fig. 2). To determine whether the differences in fluorescence intensity reflected variations in vector copy number (VCN), we quantified VCNs by ddPCR targeting the *GFP* transgene in T and NK cells transduced with SRV2 RV or RD114 RV encoding a *GFP* gene. The VCNs showed a trend consistent with the fluorescence levels, with SRV2 RV transduced T and NK cells exhibiting slightly higher VCN values (Fig. 2e, j). In addition, effective MOI was estimated from the percentage of GFP-positive cells using the Poisson formula, *m* = -ln (1 - *P*), and the calculated values are provided in Supplementary Table 4. Under matched physical titers, SRV2 RV consistently showed a higher effective MOI than RD114 RV, further supporting the superior transduction efficiency of SRV2 RV.

When we compared the transduction efficiency of SRV2 RV with that of VSV-G LV, which is widely used in CAR-T cell manufacturing (Supplementary Fig. 1, Supplementary Table. 4), SRV2 RV showed a significantly higher transduction efficiency than VSV-G LV in T, NK, and B cells. In conclusion, pseudotyping with the SRV2 ENV confers enhanced transduction efficiency and represents a promising strategy for gene delivery into immune cells.

### Only SRV2 RV exhibits efficient gene transduction among all SRV RVs

**Fig. 3:**
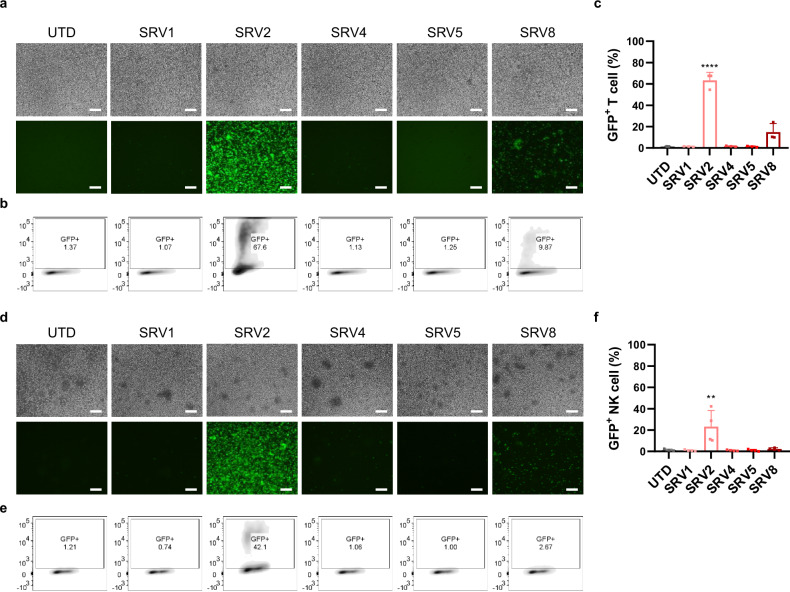
Only SRV2 RV successfully transduce genes into T cells. Retroviruses pseudotyped with SRV1, 2, 4, 5, or 8 ENVs were produced. An equal number of viral particles were used to transduce *GFP* genes into an equal number of T cells (**a**–**c**) or NK cells (**d**–**f**). Cell imaging and FACS samplings were performed 3-5 days post-transduction for T cells and 6–8 days post-transduction for NK cells. **a**, **d** The scale bar represents 10 μm, and the image was acquired at 10× magnification. 50 ms exposure for bright-field and 100 ms exposure for fluorescence field. **b**, **e** GFP fluorescence detected by Flow cytometry was analyzed as density plot. Gating strategies are provided in Supplementary Fig. 6. The proportion of GFP-positive cells shown in panels b and e was quantified in panels c and f, respectively. Data are mean ± s.d. (T, *n* = 3 donors per group; NK, *n* = 4 donors per group). Statistical analysis was performed using ordinary one-way ANOVA with Tukey’s post-hoc test. **** or ** indicates *P* < 0.0001 or *P* < 0.01 compared to all other groups. Exact *P* values for the indicated comparisons in panel f were as follows: UTD vs SRV2, *P* = 0.0013; SRV1 vs SRV2, *P* = 0.0010; SRV2 vs SRV4, *P* = 0.0010; SRV2 vs SRV5, *P* = 0.0011; and SRV2 vs SRV8, *P* = 0.0020. Source data are provided as a Source Data file.

To date, eight serotypes of SRV have been identified^22,23^. We sought to determine which SRV ENV is the most potent in pseudotyping retroviruses for gene transduction into T and NK cells. To this end, we produced SRV RVs carrying a fluorescent reporter gene pseudotyped with SRV1, SRV2, SRV4, SRV5, and SRV8 ENVs (Supplementary Fig. 2a). The efficiency of gene transduction into T and NK cells was evaluated using fluorescence intensity as a readout (Fig. 3). Interestingly, only SRV2 RV exhibited robust gene transduction in T (Fig. 3a–c) and NK (Fig. 3d-f) cells, while the other SRV RVs failed to achieve efficient transduction. SRV8 RV demonstrated limited transduction efficiency in both T and NK cells compared to SRV2 RV.

To investigate why SRV2 RVs exhibited higher transduction efficiency than other SRV serotype RVs in T and NK cells, we referred to previous findings from related viral systems. Studies have shown that differences in AAV capsid structures among serotypes result in distinct bio-distribution profiles^24^ and tissue specificity^25^, and that temperature and pH sensitivity contribute to genotype-dependent tissue tropism in Enterovirus D^26^. Based on these observations, we hypothesized that variations in the amino acid sequences of SRV ENV could also affect cell- or tissue-specific transduction efficiency. To test this hypothesis, SRV1, SRV2, SRV4, SRV5, and SRV8 pseudotyped RVs carrying a fluorescent reporter gene were employed to transduce into A549 (lung), HCT-8 (intestine), HeLa (cervix), and HepG2 (liver) cell lines (Supplementary Fig. 2b). No distinct cellular specificity was observed among the SRV serotypes. However, SRV2 RV exhibited the highest transduction efficiency in all tested cell lines. In conclusion, among all tested SRV ENV, only SRV2 ENV successfully pseudotyped retroviruses for gene transduction into T and NK cells.

### All SRV glycoproteins failed to pseudotype lentivirus for gene transduction into immune cells

**Fig. 4:**
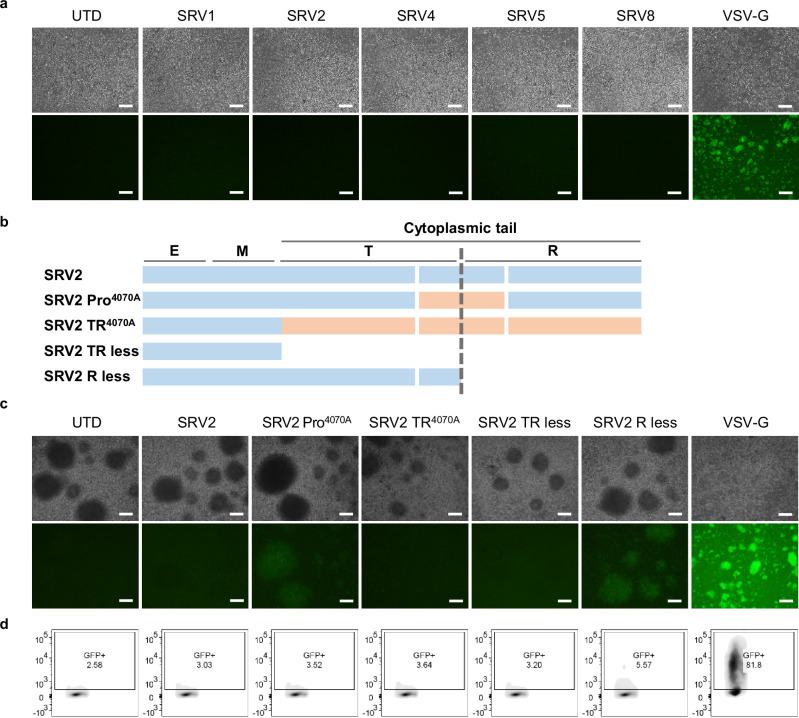
Lentiviruses pseudotyped with SRV ENV failed to transduce genes into T cells. Lentivirus pseudotyped with SRV1, 2, 4, 5, and 8 ENVs or modified SRV2 ENVs were produced. An equal number of viral particles were used to transduce *GFP* genes into an equal number of T cells (**a**) Lentiviruses pseudotyped with SRV1, 2, 4, 5, 8 ENVs, or VSV-G were infected into T cells. Cell images were taken 3-5 days after gene transduction (10× objective; scale bar, 10 µm; 50 ms exposure for both channels). Images are representative of three independent experiments with similar results. **b** Schematic representation of SRV2 wild-type ENV and cytoplasmic tail-region mutants. **c**, **d** Lentiviral vectors encoding *GFP* and pseudotyped with SRV2 wild-type ENV, SRV2 Pro^4070A^, SRV2 TR^4070A^, SRV2 TR less, or SRV2 R less ENVs were used to transduce T cells. **c** Cell images were taken 3-5 days after gene transduction (10× objective; scale bar, 10 µm; 50 ms exposure for bright field; 100 ms exposure for fluorescence images). Images are representative of three independent experiments with similar results. **d** GFP fluorescence detected by Flow cytometry was analyzed as density plot. Gating strategies are provided in Supplementary Fig. 6.

VSV-G has a broad tropism, but has a well-documented drawback of being toxic to virus producing cell lines^27,28^. In contrast, SRV2 ENV didn’t exhibit such toxicity to producer cells (Fig. 1c). Previous studies have demonstrated that retroviral ENVs can be utilized for pseudotyping lentiviruses to improve transduction efficiency in specific hematopoietic cell types, including NK cells^29–31^. Therefore, we aimed to determine whether lentiviruses pseudotyped with the SRV ENV are capable to transduce *GFP* genes into immune cells (Fig. 4a). Lentiviruses pseudotyped with SRV1, 2, 4, 5, or 8 were produced in 293 T cells, and infected into T cells. The results showed that none of the SRV pseudotyped lentiviruses achieved successful gene transduction into T cells.

In an effort to increase the gene transduction efficiency, we modified the cytoplasmic domain (CT) of SRV2 ENV. A characteristic feature of type C retroviral ENV is that the C-terminal region of their cytoplasmic tail, known as the R peptide, is cleaved by the viral protease during or shortly after viral budding. This cleavage is essential for conferring full activity to the ENV^28,32–34^. This feature is not exclusive to type C retroviruses and has been observed in other retroviruses, such as the Mason-Pfizer monkey virus (SRV3)^35^. Previous studies have shown that replacing the cytoplasmic tail region of retroviral ENV with that of Murine Leukemia Virus (MLV, 4070 A) ENV enhances the infectivity of lentivirus with retroviral ENV^34,36,37^. Based on these reports, we constructed 4 different SRV2 ENV variants (Fig. 4b). In SRV2 Pro^4070A^, the protease cleavage domain in SRV2 ENV was replaced with that of 4070 A. In SRV2 TR^4070A^, the TR domain in SRV2 ENV was replaced with that of 4070 A. SRV2 TR less or SRV2 R less lacks TR or R domain, respectively. *GFP* gene carrying lentiviruses pseudotyped with these modified SRV2 ENVs were infected into T cells (Fig. 4c-d). The results demonstrated that SRV2 Pro^4070A^ and SRV2 R less improved the gene transduction compared to wild-type SRV2. However, their transduction efficiency remained significantly lower compared to lentiviruses pseudotyped with VSV-G. Thus, we concluded that pseudotyping lentivirus with the SRV2 ENV is not suitable for gene transduction into immune cells. Thus, we have decided to focus solely on SRV2 RV in the following sections.

### Establishing the optimized protocol for producing SRV2 RV

High viral titer is critical for achieving efficient gene delivery^38–40^. Factors such as the DNA ratios of viral plasmids transfected into 293 T cells, and codon-usage bias of DNA sequences can significantly influence viral titer^29,41,42^. To optimize the production of high-titer SRV2 RV, we systematically investigated these factors (Supplementary Fig. 3). First, we tested varying ratios of transfer, packaging, and envelope plasmid to compare transduction efficiencies. The total DNA amount was fixed at 30 μg, while the envelope DNA amounts were adjusted to 12, 9, 6, or 3 μg. Transfer and packaging DNA amounts were modified accordingly to create 12 distinct ratio combinations (Supplementary Fig. 3a). Noticeable cytotoxicity or morphological damage wasn’t observed in producer cells during viral production, indicating that an increased DNA input of any specific plasmid did not cause apparent cell damage. Moreover, qRT-PCR analysis revealed that the physical titers of SRV RVs produced with different plasmid ratios were comparable. *GFP* gene was transduced into T cells with SRV2 RVs produced from these varying DNA ratios (Supplementary Fig. 3b). Among the groups with an equal amount of envelope plasmid, the combinations in which the packaging (gag-pol) and transfer plasmids were added at comparable levels—conditions (2), (5), (8), and (11)—exhibited markedly higher fluorescence intensity, with greater amounts of envelope plasmid further enhancing the transduction. Given that an appropriate Gag: Gag-Pol ratio is known to be critical for the formation of infectious particles^43–45^, these results suggest that infectious viral particles carrying the transgene were efficiently produced under conditions that allowed sufficient expression of Gag and Gag-Pol together with comparable levels of transfer RNA. Among the tested combinations, the 12:12:6 (T:E:P) ratio yielded the highest expression of the fluorescent reporter gene.

Next, we evaluated whether codon optimization could enhance the infectivity of SRV2 RV. Codon-usage bias significantly affects gene expression and functionality^46–48^, and codon optimization can often improve expression levels^49,50^. Using three codon-optimized SRV2 ENV genes (CO-a, CO-b, CO-c), we produced SRV2 RV and infected T and NK cells (Supplementary Fig. 3c-f). Unexpectedly, pseudotyped retroviruses with wild-type SRV2 nucleotide sequence showed the highest transfection efficiency. SRV2 ENV codon optimization decreased the transduction activity of SRV2 RVs. Based on these findings, we concluded that the wild-type SRV2 ENV nucleotide sequence is optimal for producing SRV2 RV and will be used for all subsequent experiments.

### CAR-T and CAR-NK cells produced by SRV2 RV outperforms those produced by RD114 RV

**Fig. 5:**
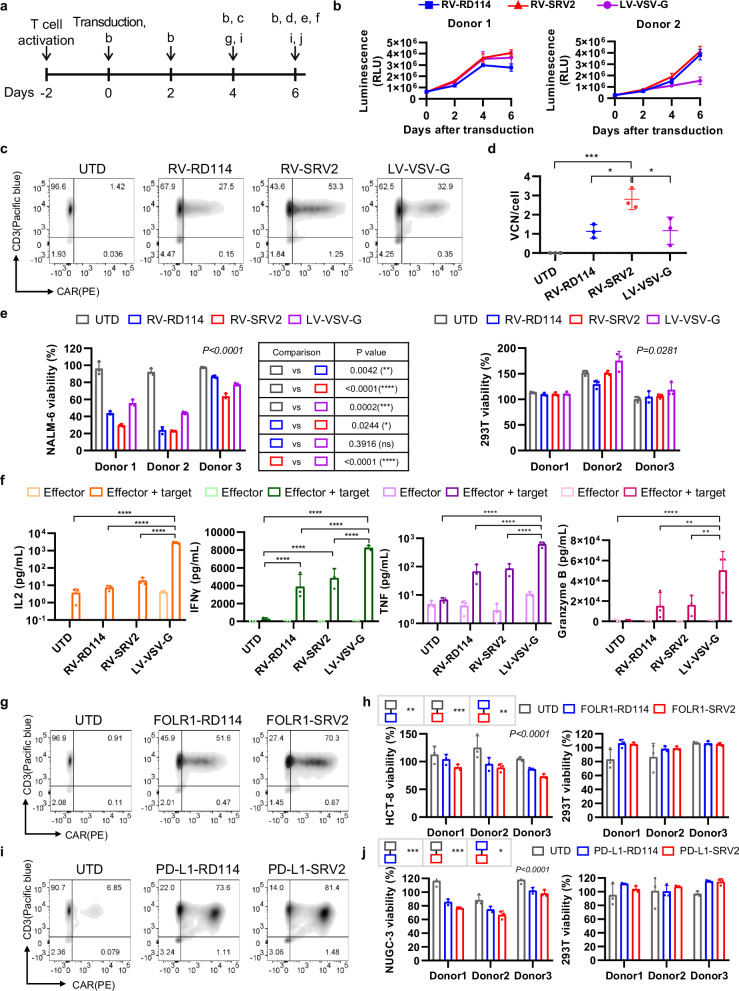
Comparison of in vitro anticancer activity of CAR-T cells generated by SRV2 RV, RD114 RV, or VSV-G LV. CD19, FOLR1, and PD-L1 CAR-T cells were generated from PBMCs of three independent donors using RD114 RV, SRV2 RV, or VSV-G LV (CD19 CAR-T: b–f; FOLR1 CAR-T: g, h; PD-L1 CAR-T: i, j). (**a**) Schematic overview of the CAR-T cell generation and in vitro evaluation workflow. (**b**–**f**) RV-RD114, CD19 CAR-T generated by RD114 RV; RV-SRV2, CD19 CAR-T generated by SRV2 RV; LV-VSV-G, CD19 CAR-T generated by VSV-G LV. (**g**,**h**) FOLR1-RD114, FOLR1 CAR-T generated by RD114 RV; FOLR1-SRV2, FOLR1 CAR-T generated by SRV2 RV. (**i**,**j**) PD-L1-RD114, PD-L1 CAR-T generated by RD114 RV; PD-L1-SRV2, PD-L1 CAR-T generated by SRV2 RV. (b) Proliferation of CD19 CAR transduced T cells from two independent donors (*n* = 3 biologically independent replicates). Luminescence was measured every two days after transduction using the CellTiter-Glo assay. (**c**, **g**, **i**) Flow cytometry analysis of CAR surface expression in CD19, FOLR1, and PD-L1 CAR-T cells. Gating strategies are provided in Supplementary Fig. 7. (**d**) Vector copy number (VCN) in genomic DNA, quantified by ddPCR targeting the CAR transgene and using *GAPDH* (2 copies) as the reference gene (*n* = 3 independent donors). (**e**, **h**, **j**) Cytotoxicity of CD19, FOLR1, and PD-L1 CAR-T cells against antigen-positive target cells (NALM-6, HCT-8, and NUGC-3, respectively) and antigen-negative 293 T cells after 24 h, measured by luminescence at an effector-to-target (E:T) ratio of 3:1 (*n* = 3 biologically independent replicates). (**f**) Cytokine secretion by CD19 CAR-T cells after 24 h coculture with NALM-6 cells at an E:T ratio of 3:1 (IL-2, IFN-γ, granzyme B, and TNF), measured by ELISA (*n* = 3 independent donors). (**b**, **d**, **e**, **h**, **j**) Gray, UTD; blue, RV-RD114; red, RV-SRV2; purple, LV-VSV-G. Data are presented as mean ± s.d. Statistical significance was determined using one-way ANOVA with Tukey’s post-hoc test (**d**, **e**, **h**, **j**) or two-way ANOVA with Tukey’s post-hoc test (**f**). *P < 0.05, **P < 0.01, ***P < 0.001, ****P < 0.0001. Exact *P* values for the indicated comparisons were as follows: in panel d, UTD vs RV-SRV2, *P* = 0.0004; RV-RD114 vs RV-SRV2, *P* = 0.0114; and RV-SRV2 vs LV-VSV-G, *P* = 0.0127; in panel e, UTD vs RV-RD114, *P* = 0.0042; UTD vs LV-VSV-G, *P* = 0.0002; and RV-RD114 vs RV-SRV2, *P* = 0.0244; in panel f (Granzyme B), RV-RD114 vs LV-VSV-G, *P* = 0.0010 and RV-SRV2 vs LV-VSV-G, *P* = 0.0013; in panel h, UTD vs FOLR1-RD114, *P* = 0.0026; UTD vs FOLR1-SRV2, *P* = 0.0002; and FOLR1-RD114 vs FOLR1-SRV2, *P* = 0.0012; in panel j, UTD vs PD-L1-RD114, *P* = 0.0006; UTD vs PD-L1-SRV2, *P* = 0.0006; and PD-L1-RD114 vs PD-L1-SRV2, *P* = 0.0160. Source data are provided as a Source Data file.

Based on the optimized SRV2 RV production protocols established in earlier experiments, SRV2 RV, RD114 RV, and VSV-G LV encoding the CD19 CAR gene were generated and used to transduce T or NK cells activated from PBMCs derived from three independent donors (Fig. 5a–f, Supplementary Figs. 4a, 5). The growth of T cells transduced with SRV2 RV, as measured by luminescence using the CellTiter-Glo assay, was maintained at a level comparable to that of T cells transduced with RD114 RV or VSV-G LV (Fig. 5b). These results suggest that the SRV2 RV vector supports enhanced cellular expansion. After viral infection into T cells, CAR expression was assessed by Flow cytometry. T cells transduced with SRV2 RV exhibited approximately 20-25% more CAR-positive T cells compared to those transduced with RD114 RV or VSV-G LV (Fig. 5c, Supplementary Fig. 4a). VCN quantified by ddPCR targeting the CAR transgene showed that SRV2 RV transduced T cells exhibited numerically higher vector copy numbers than RD114 RV or VSV-G LV transduced T cells. (Fig. 5d). CD19 CAR-T cells generated with SRV2 RV showed enhanced cytotoxic activity against CD19 positive NALM-6 cells compared with those generated using RD114 RV or VSV-G LV across three independent donors (Fig. 5e). These findings indicate that SRV2 RV is capable of generating CAR-T cells with superior antitumor activity compared to other pseudotyped viruses. Cytotoxic T cells secrete TNF and IFNγ to mediate target cell recognition and killing, and CAR-T cells eliminate tumor cells primarily through the release of perforin and granzymes^51^. Because cytokine secretion is also essential for the cytotoxic activity of CAR-T cells, we additionally assessed cytokine production (Fig. 5f). Analysis of cytokine release showed that CAR-T cells generated using SRV2 RV or RD114 RV exhibited comparable cytokine secretion profiles. In contrast, CAR-T cells generated with VSV-G LV displayed a markedly higher cytokine release than those induced by SRV2 RV or RD114 RV.

To determine whether the enhanced transduction efficiency of SRV2 RV extends to other CAR-T cells, we generated folate receptor 1(FOLR1)-CAR and PD-L1-CAR retroviruses pseudotyped with either SRV2 or RD114 ENV and transduced CAR genes into primary T cells derived from three independent donors (Fig. 5a, g-j; Supplementary Fig. 4b-c). HCT-8 is FOLR1 positive cell, and NUGC-3 is PD-L1 positive cell. 293 T is negative both for FOLR1 and and PD-L1. Across all donors, SRV2 RV transduced cells exhibited ~20% (FOLR1-CAR, Fig. 5g) and ~8% (PD-L1-CAR, Fig. 5i) higher CAR expression compared to cells transduced with RD114 RV. Consistent with this enhanced expression, SRV2 RV engineered CAR-T cells demonstrated superior target cell lysis in both CAR systems (Fig. 5h, j).

In NK cells, we also evaluated the performance of SRV2 RV (Supplementary Fig. 5). SRV2 RV transduction resulted in approximately 5-12% more CAR-positive NK cells compared to RD114 RV (Supplementary Fig. 5b). VCN quantified by ddPCR targeting the CAR transgene showed that SRV2 RV transduced NK cells exhibited higher vector copy numbers than RD114 RV transduced NK cells (Supplementary Fig. 5c). In target cell cytotoxicity assay, CD19 CAR-NK cells produced by SRV2 RV exhibited superior cytotoxicity against CD19 positive cells compared to CAR-NK cells produced by RD114 RV (Supplementary Fig. 5d). Additionally, CD19 CAR-NK cells produced by SRV2 RV secreted higher levels of cytokines in response to CD19 positive cells (Supplementary Fig. 5e).

In conclusion, CAR-T and CAR-NK cells produced by SRV2 RV demonstrated superior in vitro anticancer activity compared to those produced by RD114 RV.

### Evaluation of the Antitumor Effects of CAR-T/SRV2 RV in vivo

**Fig. 6:**
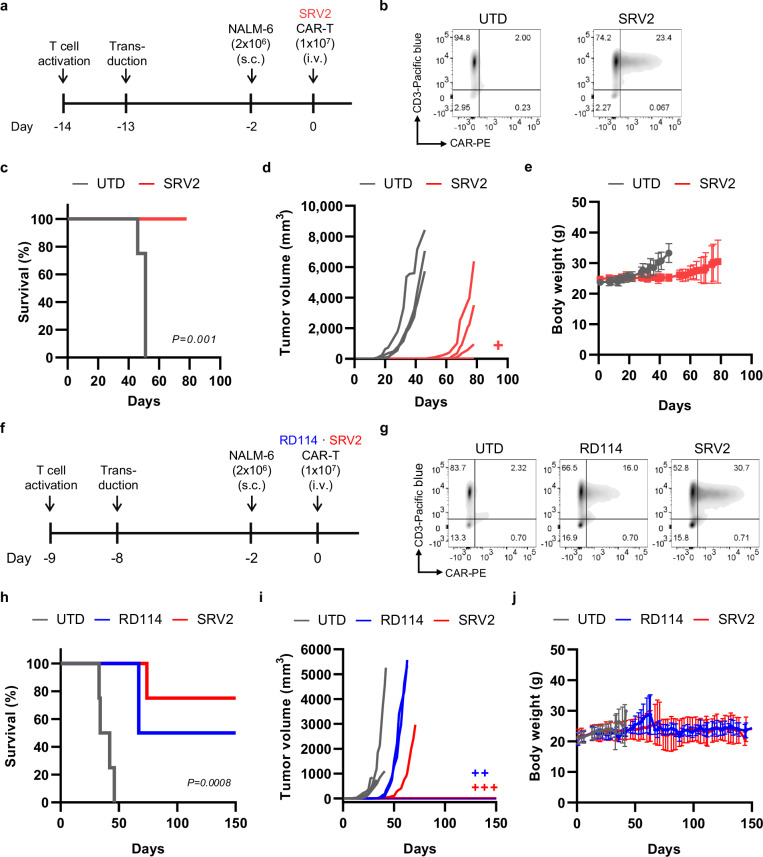
Comparison of in vivo antitumor activity of CAR-T cells generated by retroviruses pseudotyped by SRV2 or RD114. (**a**, **f**) Scheme of in vivo experiment for CAR-T cells generated by SRV2 RV or RD114 RV. Six- to eight- week-old female NSG mice were engrafted with 2×10^6^ NALM-6 cells subcutaneously followed by the treatment of 1×10^7^ CD19 CAR-T cells via i.v. (*n* = 4 mice per group). (**b**, **g**) FACS analysis showing the CAR expression of CAR-T cells produced by SRV2 RV or RD114 RV. Gating strategies are provided in Supplementary Fig. 7. (**c**, **h**) Mice survival was monitored over time and analyzed by Kaplan-Meier survival curves of mice bearing a NALM-6 tumor. Statistical differences were determined by log-lank test. (**d**, **i**) Tumor volume was measured after the injection of CAR-T cells in mice. Tumor growth was monitored over time, and tumor volume was calculated using the formula, length × width² × 0.5. Mice that did not develop tumors by the end of the study were censored and indicated by plus symbols at the right end of the x-axis in group-matched colors. (**e**, **j**) Mice body weight was measured after the injection of CAR-T cells in mice (*n* = 4 mice per group). Error bars represent mean±s.d. UTD, untransduced T cells; SRV2, CAR-T cells generated by SRV2 RV; RD114, CAR-T cells generated by RD114 RV. Across all panels, gray indicates UTD, blue indicates RD114, and red indicates SRV2. Source data are provided as a Source Data file.

Following the in vitro results demonstrating the excellent anticancer activity of CAR-T cells produced by SRV2 RV, we sought to evaluate their antitumor efficacy in vivo (Fig. 6a-e). NALM-6 cells were subcutaneously (s.c.) injected into NSG mice, and CAR-T cells were administered intravenously (i.v.). Mouse survival, tumor volume, and body weight were subsequently monitored (Fig. 6c-e). In the UTD mice, tumors began to grow around day 10 post-engraftment, and all mice succumbed to the tumor by day 46. In contrast, tumor growth in the CAR-T transduced with SRV2 RV (CAR-T/SRV2 RV) group was significantly delayed, with tumors starting to grow around day 60. Remarkably, all CAR-T/SRV2 RV treated mice survived up to day 78 (Fig. 6c-d). Notably, in one of the four mice treated with CAR-T/SRV2 RV, no tumor growth was observed throughout the study.

To compare the antitumor efficacy of SRV2 RV and RD114 RV generated CAR-T cells in vivo, NSG mice were subcutaneously (s.c.) injected with NALM-6 cells and subsequently infused intravenously (i.v.) with either CAR-T/SRV2 RV, CAR-T transduced with RD114 RV (CAR-T/RD114 RV) or UTD T cells (Fig. 6f–j). Tumor growth, body weight, and overall survival were monitored thereafter (Fig. 6h–j). In UTD mice, tumors became palpable around day 13 post-engraftment, and all mice succumbed to disease by day 42. CAR-T/RD114 treated mice exhibited delayed tumor outgrowth, with tumors emerging in 2 of 4 mice around day 33 and resulting in mortality by day 63, whereas the remaining 2 mice remained tumor-free through the end of the study. Notably, CAR-T/SRV2 RV treated mice demonstrated further prolonged tumor control, with only 1 of 4 mice developing tumors at approximately day 41 and succumbing at day 71, while the remaining 3 mice remained tumor-free for the duration of the experiment.

Together, these results indicate that CAR-T cells produced with SRV2 RV confer superior in vivo antitumor activity compared to RD114 RV generated CAR-T cells, consistent with our in vitro observations. SRV2 RV exhibited enhanced gene transduction efficiency in T cells compared with RD114 RV, which consequently resulted in improved tumor control in vivo and led to better survival outcomes.

## Discussion

In this study, we present the successful use of SRV2 RV for efficient gene transduction to produce CAR-T and NK cells. Pseudotyping is a process applicable only to enveloped viruses, such as lentivirus, retrovirus, and rabies virus, and is used to expand the cellular tropism. Pseudotyped viruses are constructed by incorporating glycoproteins from other enveloped viruses into their viral particles. The first retroviral vector containing heterologous glycoproteins was the GP160 pseudotyped HIV-1 based vector particle^52^. VSV-G, which is widely used in gene therapy for its broad cellular tropism, was efficiently incorporated into HIV-1 vector particles in 1996^53,54^. RD114 RV has also been widely used for producing CAR-based immune cells. Recently, BaEV glycoprotein pseudotyped lentiviruses demonstrated excellent gene transduction into hematopoietic cells, including B, T, and NK cells^55^. Both RD114 and BaEV glycoproteins utilize ASCT2 as their cellular receptor^15,21^. Simian type D retroviruses, such as SRV, also use ASCT2 for cell entry^14^. However, their potential for pseudotyping retroviral or lentiviral vectors to target immune cells has not been explored until now. Thus, in this study, we evaluated the utility of SRV2 pseudotyped viral vectors for gene transduction into immune cells.

We found that SRV2 glycoproteins are non-toxic to virus-producing cells, such as 293 T cells, a property that is essential for maintaining high and stable yields of retroviral vectors (Fig. 1c). Notably, SRV2 RV was achieved higher titers than RD114 RV, indicating that SRV2 ENV not only avoid cytotoxicity but also support more robust and efficient vector production (Fig. 1d). SRV2 RV demonstrated efficient gene transduction into immune cells, including T, NK, and B cells, outperforming RD114 RV (Fig. 2). In T and NK cells, SRV2 RVs yielded higher vector copy numbers than RD114 RVs (Fig. 2e, j), suggesting that the increased transgene expression was primarily attributable to enhanced transduction efficiency and the resulting increase in transgene copy number per cell, rather than to an intrinsic property of the SRV2 ENV that directly enhances transgene expression. This interpretation is consistent with previous findings showing that higher vector copy number is associated with improved transduction efficiency and enhanced functional attributes of retrovirally engineered T cell products^56^.

However, lentiviruses pseudotyped with SRV failed to transduce genes into immune cells (Fig. 4). This limitation likely stems from challenges associated with incorporating retroviral glycoproteins into lentiviral particles^28^. For example, GALV or RD114 pseudotyped lentiviral vectors exhibit significantly low infectivity because their glycoproteins are not cleaved by lentiviral protease, preventing effective packaging of lentiviral vectors such as HIV and simian immunodeficiency virus (SIV)^37,40,57^. Modifying R peptides in retroviral glycoproteins can improve their compatibility with lentiviral proteases^31,37,58,59^. However, our efforts to enhance SRV2 lentiviral infectivity through similar modifications were unsuccessful. Thus, we concluded that SRV2 is incompatible with lentiviral pseudotyping. Several factors may explain why lentiviral pseudotyping was not efficiently achieved despite extensive modification of the SRV2 glycoproteins. According to Tomas et al.^37^, replacing the protease recognition region of RD114 or GALV with the HIV-1 natural sequence resulted in more efficient protease cleavage and higher infectious particle output, whereas substitution with the MLV motif was considerably less effective. Even with this improvement, VSV-G still yielded substantially higher functional titer. Because our SRV2 constructs incorporated the MLV-derived protease recognition site, it is likely that protease processing remained suboptimal, leading to markedly lower infectivity relative to VSV-G pseudotypes. In addition, ENV packaging into HIV-1 particles requires a precise interaction between the HIV-1 Matrix domain (MA) and the cytoplasmic tail (CT) of ENV^60^. Thus, although we introduced TR modifications to enhance this compatibility, these changes may not have fully restored MA–CT binding efficiency, ultimately limiting the pseudotyping performance of SRV2 in the lentiviral system.

We further examined the pseudotyping potential of different SRV glycoproteins. 8 subtypes of SRV were isolated from macaques^22,23^. SRV1 infection causes hematological abnormalities such as anemia and granulocytopenia^7^. SRV2 can induce tumor or other immune suppressive symptoms^61^. SRV3, which is Mason Pfizer Monkey Virus, shares 83% identity with SRV1^62^. SRV4 and 5 induce histiocytic inflammation and dysfunction of B cells, respectively^63^. SRV8 was identified recently, and not reported regarding pathogenicity and immunogenicity^23^. In this study, we evaluated whether the SRV1, 2, 4, 5, and 8 ENV could be used for retroviral pseudotyping. Only SRV2 RV demonstrated robust and efficient gene transduction into immune cells as well as multiple cell types derived from different tissues, with SRV8 showing modest but clearly detectable activity, whereas all other serotypes exhibited negligible or no transduction under the same conditions (Fig. 3; Supplementary Fig. 2). These findings highlight SRV2 as the most promising glycoprotein for pseudotyping retroviruses among SRV subtypes. Because amino acid variations within ENV regions that mediate host-receptor binding and membrane fusion are known to alter fusion activity and viral infectivity^64–66^, differences in the ENV sequences among SRV serotypes could plausibly contribute to the observed variation in viral infectivity.

To develop better SRV2 RV, we made codon optimized SRV2 sequences (Supplementary Fig. 3). Codon optimization is a well-known strategy to improve the infectivity of viral vectors by increasing the production of functional and structural viral proteins^67,68^. However, in our study, the codon optimization strategy didn’t improve the infectivity at all. Codon optimization doesn’t always ensure the improvement due to many reasons^69^.

Finally, we manufactured CAR-T and CAR-NK cells with SRV2 RV or RD114 RV. CAR-T and CAR-NK cells produced using SRV2 RV exhibited superior characteristics compared to those produced using RD114 RV (Fig. 5; Supplementary Figs. 4, 5). CD19, FOLR1, PD-L1 CAR-T cells produced by SRV2 RV had a higher percentage of CAR-positive cells and demonstrated enhanced cytotoxicity against antigen-positive cells. Notably, cytokine secretion did not differ between CAR-T cells generated using RD114 RV and SRV2 RV. This observation aligns with recent studies showing that CAR-T cells derived from different donors can exhibit substantial donor-to-donor variability in cytokine secretion profiles, even when generated using the same CAR construct^70,71^. Consistent with this, our data indicate that cytokine production alone may not fully capture functional differences between the two CAR-T cells. Importantly, however, the direct functional readout of target cell cytotoxicity showed a consistent and reproducible difference across multiple donors, suggesting that cytotoxic activity represents a more robust and biologically relevant measure of CAR-T cell function in this context. Similarly, CD19 CAR-NK cells produced with SRV2 RV exhibited improved gene transduction and greater target cell cytotoxicity compared to those produced using RD114 RV. RD114 RV are currently the most widely used vectors for manufacturing CAR-T and CAR-NK cells due to their high transduction efficiency in immune cells. However, our results showed that SRV2 RV outperform RD114 RV in vitro. Importantly, we demonstrated that CAR-T cells generated with SRV2 RV exhibited superior antitumor activity in vivo compared with those produced using RD114 RV (Fig. 6). These findings suggest that SRV2 RV could be a practical and effective option for manufacturing CAR-T or CAR-NK cells in clinical settings.

Nevertheless, several limitations should be acknowledged. Although previous studies have suggested that vector integration near proto-oncogenes can contribute to leukemogenesis^72^, the present study did not assess the integration-site preference or potential genotoxic risk of SRV2 RV. Given that the balance between safety and efficacy is a key consideration for the clinical translation of any new retroviral vector system, further studies will be required to define the safety profile of SRV2 RVs in greater detail. In addition, the in vivo experiments were conducted with relatively small cohort sizes, which may limit the robustness of the observed antitumour effects. Larger and more adequately powered in vivo studies will therefore be needed to further validate the therapeutic potential of SRV2 RV based CAR immune cells. In conclusion, our study identifies SRV2 glycoprotein as a promising pseudotyping ENV for retroviral vectors that enables efficient gene transfer into T and NK cells. By supporting efficient vector production and enhanced transduction, SRV2 RV may serve as a useful platform for improving the manufacture of gene- and cell-based therapeutics and expanding their potential applications in cancer immunotherapy.

## Methods

### Ethics statement

Human peripheral blood mononuclear cells (PBMCs) were obtained from healthy donors who provided written informed consent according to procedures approved by the Institutional Review Board of the Korea National Institute for Bioethics Policy (Approval no. P01-201607-31-003). All experimental protocols using PBMCs were approved by IRB. NOD-SCID IL2R γ null (NSG) mice were purchased from Charles River Laboratories Japan, Inc. All procedures are approved by the Laboratory Animal Care and Use Committee of the Korea Research Institute of Chemical Technology and were conducted in accordance with the Institute for Laboratory Animal Research Guide for the Care and Use of Laboratory Animals. All efforts were made to minimize animal suffering.

### Viral vector construction

The nucleotide sequences of the ENVs of SRV1, SRV2, SRV4, SRV5, and SRV8 were retrieved from NCBI. Genes were synthesized by Twist Bioscience and cloned into expression vectors. The *EGFP*, CD19 CAR, FOLR1 CAR, and PD-L1 CAR gene were cloned into SFG vectors individually. Primers were synthesized by Macrogen, and all primers used in the cloning procedures are listed in Supplementary Table 1. PCR was performed using the Phusion High-Fidelity DNA Polymerase kit (NEB, M0530L) with the following steps: denaturation at 95°C for 30 seconds, annealing for 30 seconds at the temperature determined by each primer’s Tm, and DNA extension at 72°C for 1 kb/min. PCR amplification was carried out for 30 cycles. Codon optimization of the nucleotide sequences was performed in the website of GeneScript (https://www.genescript.com), Twist Bioscience (https://www.twistbioscience.com), and IDT DNA. Codon optimized SRV2 ENV genes were synthesized by Twist Bioscience and cloned into expression vectors.

### Production and titration of pseudotyped lentiviruses or retroviruses

For retrovirus production, pEQ-PAM-3E packaging DNA and RD114 envelope DNA^16^, kindly provided by Prof. Dario Campana (National University Cancer Institute Singapore), were used together with SFG vector (Addgene, 22493)-based transfer DNA. In some experiments, SRV envelope DNA cloned from the RD114 vector was used in place of RD114 envelope DNA. For lentivirus production, psPAX packaging DNA (Addgene, 12260), VSV-G (Addgene, 12259), BaEV-TR^73^, kindly provided by Prof. Duck Cho (Samsung medical center), and SRV2 PRO^4070A^, SRV2 TR^4070A^, SRV2 TR less, or SRV2 R less envelope DNA were used together with pCDH vector (System Biosciences, CD500B-1)-based transfer DNA. A total of 8 × 10^6^ 293 T cells were seeded in a 100 mm dish (Falcon, 353003) one day before transfection. The next day, transfer, envelope, and packaging DNAs were mixed at appropriate ratios and transfected into 293 T cells using 2 M CaCl_2_ (Sigma-Aldrich, C5670) and 2X HBS (Biosesang, H2089-050-00). The viral supernatant was collected and filtered through a 0.45 μm PES filter (Millipore, SLHPR33RB). Viral RNA was extracted for titration using the NucleoSpin RNA Virus kit (Macherey-Nagel, 740956.240). Viral titers were measured using the Retro-X qRT-PCR Titration Kit (Clontech, 631453) or the Lenti-X qRT-PCR Titration Kit (Clontech, 631235).

### PBMC isolation

All methods using blood samples were performed in accordance with institutional biosafety guidelines. A total of 500 mL of blood was mixed with an equal volume of PBS (Biosesang, PR2007-000-00) containing 2% Fetal Bovine Serum (FBS, Gibco, 16000-044). Then, 30 mL of the blood mixture was slowly layered onto 15 mL of Lymphoprep (STEMCELL, 18061) in a 50 mL conical tube (SPL, 50050). The layered solution was centrifuged at 800 x g for 20 minutes at room temperature without brake. After centrifugation, the top plasma layer was discarded, and the PBMC layer between the plasma layer and Lymphoprep solution was collected into a new tube. The collected PBMCs were washed twice with PBS containing 2% FBS and centrifuged at 300 × *g* for 8 minutes at room temperature. After counting, PBMCs were mixed with freezing medium (90% FBS and 10% DMSO (Sigma-aldrich, D2438)), divided into equal aliquots, and cryopreserved in liquid nitrogen tank.

### Cell culture

All cultured cells were maintained in a humidified incubator at 37°C with 5% CO₂. 293 T (ATCC, CRL-1573), HeLa (ATCC, CCL-2), and HepG2 (ATCC, HB-8065) cells were cultured in DMEM with high glucose (HyClone, SH30243.01) supplemented with 10% FBS. NALM-6 (ATCC, CRL-3273), A549 (ATCC, CCL-185), HCT-8 (ATCC, CCL-244), and NUGC-3 (JCRB, JCRB0822) cells were cultured in RPMI1640 (HyClone, SH30027.01) supplemented with 10% FBS. Cryopreserved PBMCs were quickly thawed in a 37 °C water bath and activated into T cells using human T-activator CD3/CD28 Dynabeads (Gibco, 11132D) and RPMI1640 medium with 10% FBS. T cells were used in experiments within 1 to 3 days after activation. T cells were cultured for 7 to 14 days, with fresh medium containing 10 ng/mL of human recombinant IL-2 (R&D Systems, 202-IL) replenished every 3 days. NK cells were isolated from PBMCs following the protocol provided by the NK Cell Isolation Kit (STEMCELL, 17955). Isolated NK cells were cocultured with X-ray-irradiated K562 feeder cells expressing membrane-bound IL-2 (mbIL2) at a 1:5 NK-to-feeder ratio. The NK cell culture medium consisted of NK MACS medium (Miltenyi Biotec, 130-114-429) supplemented with 5% human serum (Sigma-aldrich, H3667), 25 ng/mL human recombinant IL-2, 25 ng/mL human recombinant IL-15 (PeproTech, 200-15), and 25 ng/mL human recombinant IL-21 (PeproTech, 200-21). Fresh medium containing the cytokines was added every 2 days. NK cells were used for experiments when they were fully activated, which typically occurred 6 to 8 days post-isolation, as indicated by the complete depletion of K562 feeder cells. B cells were isolated from PBMCs following the protocol provided by the B cell isolation kit (STEMCELL, 17914) and cultured using the Human B Cell Expansion kit (STEMCELL, ST100-0645). After isolation, B cells were used in experiments once their maturation was confirmed by the expression of CD19 (positive), CD3 (negative), and CD80 (positive) markers using Flow cytometry.

### Viral vector-mediated cell transduction

Activated T, NK, and B cells were counted, and equal numbers of cells were distributed into tubes containing either pseudovirus or DMEM (untransduced control, UTD). Polybrene (hexadimethrine bromide, Sigma-Aldrich, H9268) was added at a final concentration of 8 μg/mL. Centrifugation was performed at 2000 × *g* for 90 minutes at room temperature (RT) for T and NK cells, 1800 × *g* for 90 minutes at RT, and 1000 × *g* for 90 minutes at RT for B cells, A549, HepG2, HeLa and HCT-8. After centrifugation, the supernatant was removed, and the cells were resuspended in their respective culture media for incubation. Because ENV dependent differences in functional infectivity preclude the use of a single nominal MOI across pseudotypes, equal amounts of viral particles were used for all transduction experiments, normalized to physical titers (viral genome copies). The cell-to-virus ratios, calculated based on physical titers at the time of transduction, were as follows: 1:10,000 for *GFP* transduced T cells, 1:20,000 for CAR transduced T cells, 1:100,000 for *GFP* transduced NK cells, 1:500,000 for CAR transduced NK cells, and 1:50,000 for *GFP* transduced B cells. Effective MOI was estimated from the percentage of GFP-positive cells using the Poisson formula, *m* = -ln (1 - *P*), where P represents the fraction of GFP-positive cells. This analysis was used to compare relative transduction efficiency across pseudotypes under matched physical titers.

### Cell imaging

Cell imaging was performed using an Eclipse TE300 microscope (Nikon) equipped with a DS-Ri1 camera (Nikon). Fluorescence was observed with a Precentered Fiber Illuminator (Nikon). Images were acquired at 4× or 10× magnification, and scale bars were added to the figures based on the resolution and magnification of the camera.

### ddPCR based Vector Copy Number (VCN) analysis

Genomic DNA (gDNA) was extracted from 1 × 10^6^ to 5 × 10^6 ^T or NK cells using the G-DEX™ IIc Genomic DNA Extraction Kit (Intron, 17231) according to the manufacturer’s instructions. The gDNA concentration was measured using a NanoDrop Lite spectrophotometer (Thermo Fisher Scientific) and stored at −20 °C until further use. Droplet digital PCR (ddPCR) was performed following the recommended guidelines from Bio-Rad. Primers and probes used to detect *GFP*, CAR, and *GAPDH* are listed in Supplementary Table 2. Each 20 µL ddPCR reaction contained ddPCR Supermix for Probes (no dUTP, Bio-rad, 1863026), 2 ng of gDNA, 900 nM of each primer, and 250 nM of each probe. Droplets were generated using a QX200 Manual Droplet Generator (Bio-Rad) and transferred to a 96-well ddPCR plate (Bio-rad, 12001925). PCR amplification was performed on a T100 thermal cycler (Bio-Rad) using the following program: 95 °C for 10 min; 40 cycles of 94 °C for 30 s, 60 °C for 1 min, and 72 °C for 30 s; followed by 98 °C for 10 min. The temperature ramp rate was set to 2 °C/s. After amplification, droplets were analyzed on a QX200 Droplet Reader (Bio-Rad) using QX Manager software (v2.1). Copy numbers for *GFP* or CAR and the reference gene *GAPDH* were quantified. Vector copy number (VCN) per cell was calculated based on the ratio of target transgene copies to *GAPDH* copies, assuming two *GAPDH* copies per diploid genome (VCN = [*GFP* or CAR copies / *GAPDH* copies] × 2).

### CAR-T cell proliferation using CellTiter-Glo luminescent assay

To evaluate the proliferation of CAR-T cells following viral transduction, we performed a luminescent ATP-based CellTiter-Glo assay (Promega, G7572). Immediately after viral transduction, 1 × 10³ cells per well were seeded into a 96-well plate. The luminescence value measured immediately after plating was defined as day 0. Cell proliferation was assessed every two days. For each measurement, an equal volume of CellTiter-Glo reagent was added to each well, mixed gently by pipetting, and incubated for 10 minutes at room temperature, protected from light, before quantification using an EnSpire Alpha Multimode Plate Reader (PerkinElmer).

### FACS analysis

Control or experimental cells (1 × 10^6^) were placed in 1.5 mL EP tubes (Axygen, MCT-150-C) and centrifuged at 1250 × *g* for 5 minutes at 4 °C. After removing the supernatant, the cells were washed twice with staining buffer (PBS containing 0.2% BSA (GenDEPOT, A0100-005) and 0.08% NaN_3_ (Sigma-aldrich, S2002)). The cells were then resuspended in 50 μL of staining buffer, and 1 μL of antibody or protein was added for primary staining, which was performed for 30 minutes. For secondary staining, if needed, the cells were washed twice and stained using the same procedure as for primary staining. After two additional washes, the cells were fixed with 250 μL of fixation buffer (PBS containing 4% paraformaldehyde (Biosesang, PC2031-050-00)) for 10 minutes, followed by two more washes and filtration through a cell strainer (Falcon, 352235). For *GFP*-expressing cells, the same centrifugation and washing steps were applied, followed by fixation with fixation buffer, two additional washes, and filtration through a cell strainer. The samples were analyzed using a FACS Canto II (BD), and data were processed with FlowJo software (v10.10.0). Antibodies used for FACS analysis are listed in Supplementary Table 3.

### Cell Cytotoxicity Assay

Positive or negative target cells expressing luciferase were counted and seeded at 1 × 10^4^ cells per well in a 96-well plate (SPL, 30096). UTD and CAR-T cells were also counted and added to each well according to the specified effector-to-target (ET) ratio. Target-only wells were used as the negative control for the assay. After coculture for 4 or 24 hours, cytotoxicity was assessed using Bright-Glo Luciferase Assay System (Promega, E2620). An equal volume of Bright-Glo reagent was added to the coculture medium in each well, mixed by pipetting, and incubated for 10 minutes protected from light before measuring luminescence using an EnSpire Alpha Multimode Plate Reader (PerkinElmer). The viability of target cells cocultured with UTD or CAR-T cells was calculated relative to the luminescence values of target only wells.

### Enzyme-Linked Immunosorbent Assay (ELISA)

UTD or CAR-T cells were cocultured with target cells at an effector-to-target (ET) ratio of 3:1. After 24 hours, the supernatants were collected and appropriately diluted for the ELISA assay. The ELISA MAX Deluxe Set (BioLegend) for IL-2 (431804), IFN-γ (430104), TNF (430204), and granzyme B (439204) was used, and the assay was performed according to the manufacturer’s protocol. Cytokine concentrations were calculated by multiplying the measured values by the dilution factor and subtracting the media-only background, based on the corresponding standard curves.

### Xenograft studies

Four- to six-week-old female NOD-SCID IL2R γ null (NSG) mice were obtained from Charles River Laboratories Japan, Inc. (JAX stock no. 005557) and maintained in a specific pathogen-free (SPF) animal facility for 2 weeks. NALM-6 cells (2 × 10^6^ cells/mouse) were engrafted subcutaneously into the right flank. Two days later, mice were randomly assigned to groups (*n* = 4 per group), and intravenously injected with either mock or CD19 CAR-T cells (1 × 10^7^ cells/mouse). Experimental and control animals were co-housed before T cell (effector) injection and housed separately after randomization. Tumor size was measured every 2–4 days using calipers, and tumor volume was calculated as length × width² × 0.5. Body weight was recorded at the same time points. In accordance with institutional ethical guidelines, tumor burden was allowed to reach approximately 10% of the mouse’s body weight in efficacy studies, and we adhered to this limit. Animals were monitored regularly for tumor progression, body weight, and general condition. Mice were euthanized by CO_2_ inhalation when predefined humane endpoint criteria were met, including interference of the tumor with normal body function, infection at the tumor site, or a body weight loss of more than 20% compared with age-matched normal controls.

### Statistical analysis

Statistical analyses were performed using GraphPad Prism 8 software (v8.0). For comparisons among more than three groups, one-way ANOVA was performed, and when a significant overall effect was detected, Tukey’s post hoc test was applied. For comparisons between two groups, normality was first assessed using the Shapiro–Wilk test, followed by an appropriate two-tailed *t*-test depending on the normality results. For the analysis of in vivo tumor size and ELISA assay, *P* values were determined using two-way analysis of variance (ANOVA). Survival differences between groups were evaluated using Kaplan–Meier survival curves and the log-rank test. *P* values less than 0.05 were considered statistically significant, with *P < 0.05, **P < 0.01, ***P < 0.001, and ****P < 0.0001.

### Reporting summary

Further information on research design is available in the Nature Portfolio Reporting Summary linked to this article.

## Supplementary information

Supplementary Information

Reporting Summary

Transparent Peer Review file

## Source data

Source Data

## Data Availability

All data are included in the Supplementary Information or available from the authors, as are unique reagents used in this Article. The raw numbers for charts and graphs are available in the Source Data file whenever possible. Source data are provided with this paper.
