# Supplementary material for: Discovery of a novel envelope protein derived from simian retrovirus 2 for pseudotyping retroviral vectors used for production of CAR immune cells
Source: Nat Commun. 2026 Apr 23;17:5643. doi: 10.1038/s41467-026-72024-4 (PMC13315792; doi:10.1038/s41467-026-72024-4)
Supplement: Supplementary file 1 — Supplementary Information [file 41467_2026_72024_MOESM1_ESM.pdf]

Supplementary Information:
Discovery of a novel envelope protein derived from simian retrovirus 2 for pseudotyping retroviral vectors used for production of CAR immune cells

Moonjung Jeun[1,2], Yeongrin Kim[1], Heung Kyoung Lee[1], Ji U Choi[1], Hye Gwang Jeong[2], and Chi Hoon Park[1,3*]

** Corresponding author: Chi Hoon Park, chpark@krict.re.kr*

Containing:
Supplementary figure. 1 to 7
Supplementary Table. 1 to 4

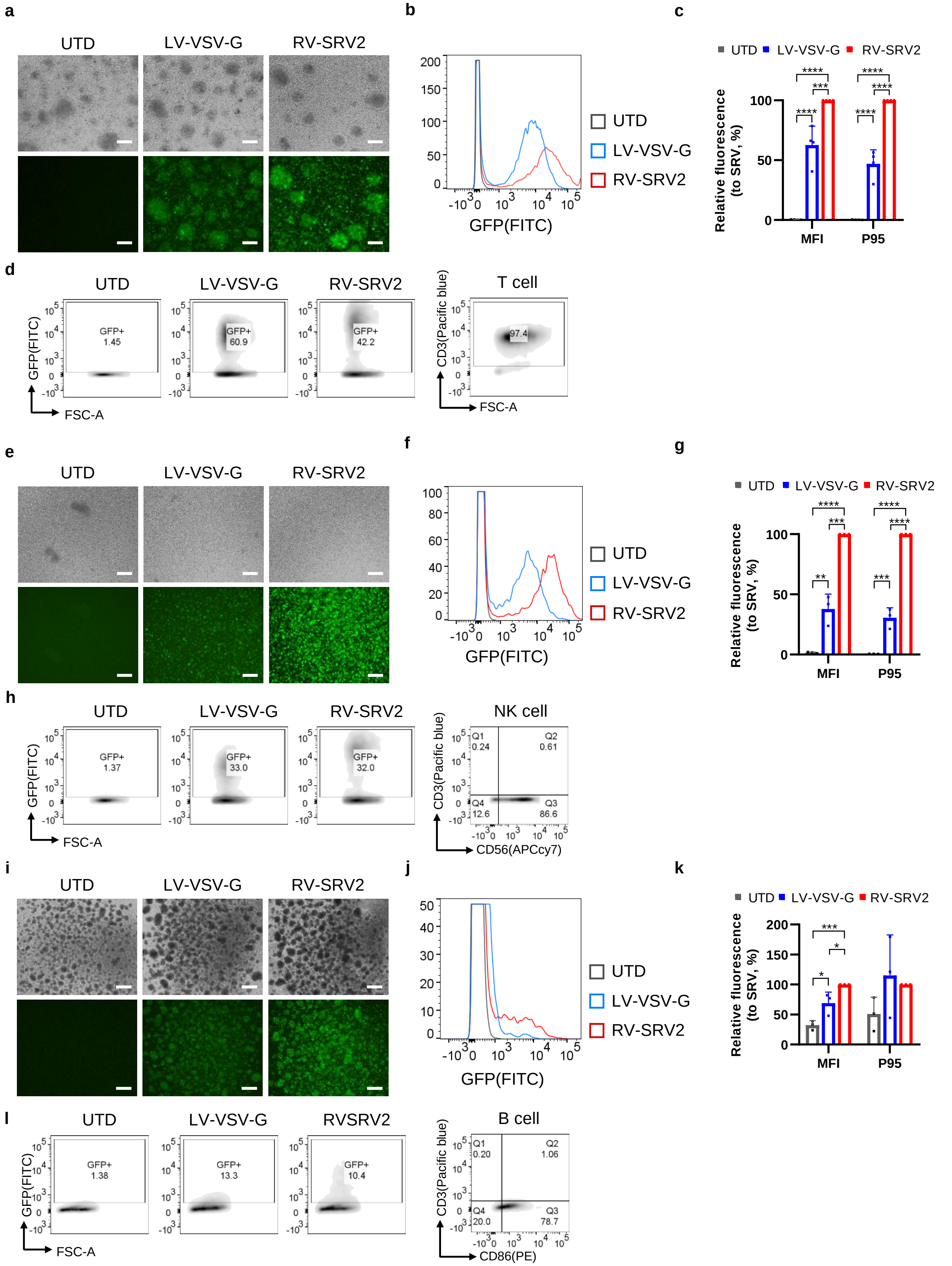

a
UTD
LV-VSV-G
RV-SRV2
b
GFP(FITC)
UTD
LV-VSV-G
RV-SRV2
c
Relative fluorescence (to SRV, %)
MFI
P95
d
GFP(FITC)
FSC-A
GFP+ 1.45
GFP+ 60.9
GFP+ 42.2
T cell
CD3(Pacific blue)
97.4
e
f
g
h
GFP+ 1.37
GFP+ 33.0
GFP+ 32.0
NK cell
Q1 0.24
Q2 0.61
Q4 12.6
Q3 86.6
CD56(APCcy7)
i
j
k
l
RVSRV2
GFP+ 1.38
GFP+ 13.3
GFP+ 10.4
B cell
Q1 0.20
Q2 1.06
Q4 20.0
Q3 78.7
CD86(PE)

Supplementary figure 1 | Enhanced Gene Delivery to Primary Human Immune Cells by SRV2 RV compared to VSV-G LV.

SRV2 RV or VSV-G LV were used to transduce GFP gene into primary human T cells (a-d), NK cells (e-h), and B cells (i-l). UTD, untransduced cells; LV-VSV-G, cells transduced with VSV-G LV; RV-SRV2, cells transduced with SRV2 RV. Imaging, flow cytometric analyses were performed 3–5 days post-transduction for T and B cells, and 6–8 days post-transduction for NK cells. (a, e, i) Representative bright-field and fluorescence images of transduced T, NK, and B cells (10×(a,e) or 4×(i) objective; scale bar, 10 µm; 50 ms exposure for both channels). (b, d, f, h, j, l) Flow cytometry histograms and density plots showing GFP expression in T, NK, and B cells transduced with SRV2 RV or VSV-G LV. Immune subsets were confirmed by lineage markers (T, $CD3^+$; NK, $CD3^-CD56^+$; B, $CD3^-CD86^+$). Gating strategies are provided in Supplementary fig. 6. (c, g, k) Quantification of GFP expression based on mean fluorescence intensity (MFI) and 95th percentile fluorescence (P95). Data are mean ± s.d. (T, n = 4 biologically independent experiments; NK, n = 3 biologically independent experiments; B, n = 3 biologically independent experiments). Statistical significance was determined by one-way ANOVA with Tukey's post-hoc test. *$P < 0.05$, **$P < 0.01$, ***$P < 0.001$, ****$P < 0.0001$. Source data are provided as a Source Data file.

a

```
SRV1      PCDCAGGYVSSPPTNSLTTVSCSTYTAYSVTNSLKWQCVSTPTTASPTHIGSCPSQCNSQ   60
SRV4      PCECKGGYVNRPPSSHVASISCGSHTAYQPTNNLKWQCVSTPKTTSGGHMGQCPSACSDK   60
SRV8      PCDCGGGYTQTQPTTYVTSISCGSHTAYSQVGSLKWLCIKAPSTPSGQHVGHCPQDCNTQ   60
SRV2      PCDCAGGYVNAAPTVYLAAVSCSSHTAYQPSDSLKWRCVSNPTLANGENIGNCPCKT---   57
SRV5      PCDCAGGIVSSPPTAYVASVSCSTHTAYQVTTSLKWQCVNFPTRSNGASAGICACEV---   57
          ** * **     *       **   ***     *** *   *        * *

SRV1      SYDSVHATCYNHYQQCTIGNKTYLTATMIRDKSPSSGDGNVPTILGNNQNLIIAGCPENK  120
SRV4      SYDSVHSTCYSSYQQCTIGNKTYFTATITGERTATIGVSNVPTVIGSGQNLISAGCPKDE  120
SRV8      SYESMHSSCYTSYQQCTLGNKTYFTAAIIKNRSPSVGQ----SVLGSSHNLITAGCH--D  114
SRV2      FKESVHSSCYTAYQECFFGNKTYYTAILASNRAPTIGTSNVPTVLGNTHNLLSAGCTG-N  116
SRV5      ILPSVHATCYSSYQTCKSNGTTYNTAKLLSARNPTMGQSNVPSVLGNSHNLISSGCDKNN  117
             * *  **  ** *     ** **          *        *   **   **

SRV1      KGQVVCWNSQPSVHMSDGGGPQDKVREIIVNKKFEELHKSLFPELSYHPLALPEARGKEK  180
SRV4      LGKTACWSATPPVHVSDGGGPQDKVREILVQKKFEELHKNLFPELSYHPLALPKARGKEK  180
SRV8      TGKTACWNTHPPVHVSDGGGPQDKVREIIVEKKLEEIQKSLFPELSYHPLALPETRGKEM  174
SRV2      VGQPICWNPKAPVHISDGGGPQDKAREIAVQKRLEEIHKSLFPELRYHPLALPKARGKEK  176
SRV5      VGKQVCWSVTPPVHMSDGGGPQDQAREILVHKKLEELQKALFPELSYHPLALPKARGKEK  177
           *   **     ** ********  *** * *  **  * ***** *******  ****

SRV1      IDAHTFDLLATVHSLLNVSSQRQLAED                                   207
SRV4      IDAQTVDILTAVHNLLNLTRPDL-AQD                                   206
SRV8      IDPQTFEILTTVHSLLNATKPDL-AQD                                   200
SRV2      IDAQTFNLLTATYSLLNKSNPNLANE-                                   202
SRV5      IDAQTLDLLATVHSLLNESRPDLTDG-                                   203
          **  *   *     ***
```

b

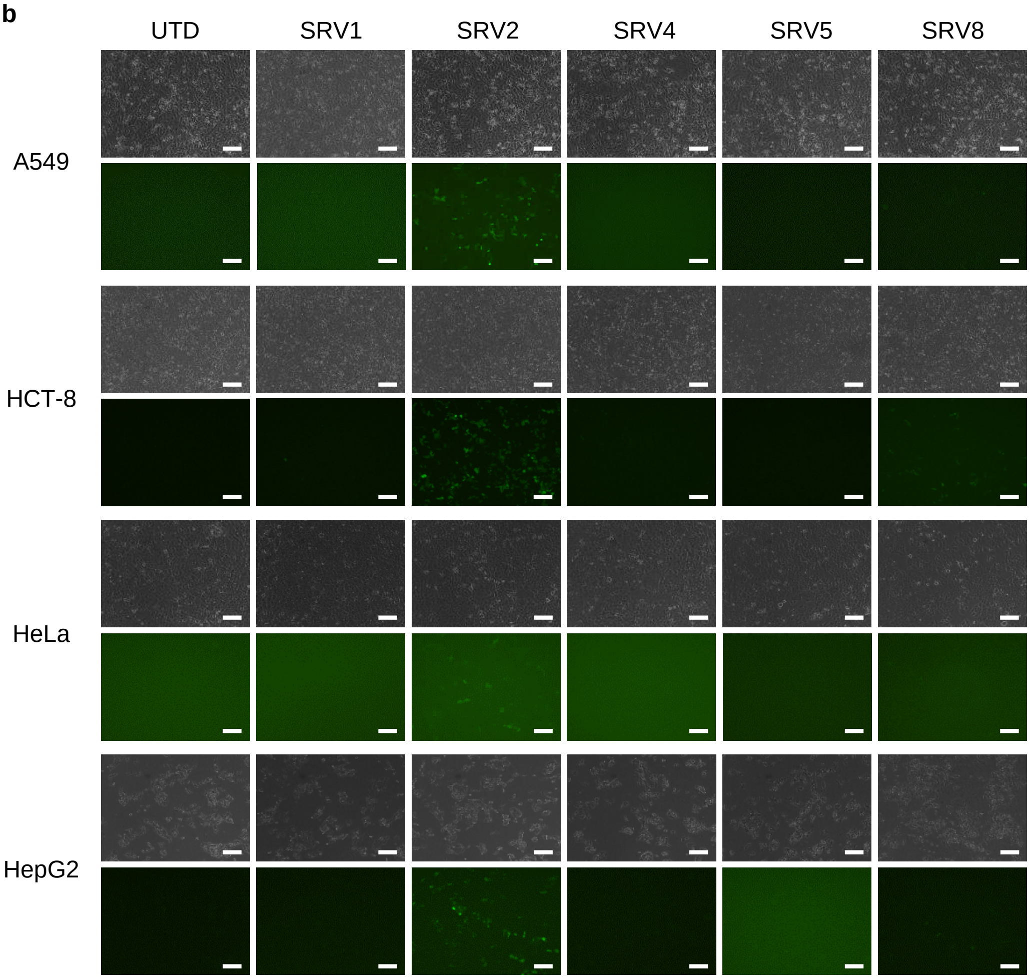

Supplementary figure 2 | Among SRV serotypes, SRV2 exhibits the highest gene-delivery efficiency across diverse tissue-derived cell types.

(a) The RBD domain sequences of SRV1 (AAA47733.1), SRV2 (AAA47563.1), SRV4 (YP_003864103.1), SRV5 (BBG56793.1), and SRV8 (YP_009305202.1) are aligned for comparison. The conserved amino acid sequences are indicated by asterisks below the alignment. (b) SRV1, 2, 4, 5, and 8 RVs carrying a fluorescent reporter gene were used to transduce A549 (lung derived), HCT-8 (intestine derived), HeLa (cervix derived), and HepG2 (liver derived) cell lines. Representative bright-field and fluorescence images of transduced cells (10× objective; scale bar, 10 μm; 50 ms exposure for both channels). Images are representative of three independent experiments with similar results.

a

Ratio	(1)	(2)	(3)	(4)	(5)	(6)
Transfer(μg)	12	9	6	16	11	5
Envelope(μg)	12	12	12	9	9	9
Packaging(μg)	6	9	12	5	10	16
**Ratio**	**(7)**	**(8)**	**(9)**	**(10)**	**(11)**	**(12)**
Transfer(μg)	18	12	6	20	14	7
Envelope(μg)	6	6	6	3	3	3
Packaging(μg)	6	12	18	7	13	20

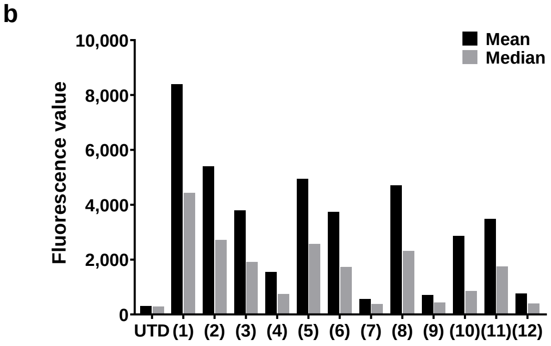

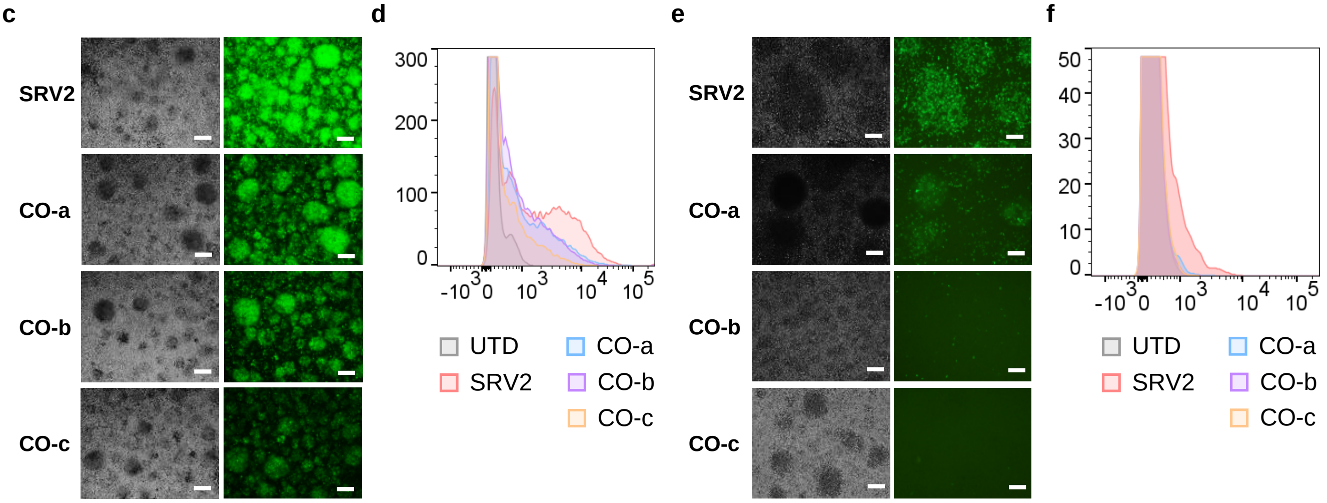

Supplementary figure 3 | Optimization of the production protocol for SRV2 RV

(a) DNA mass ratios of viral plasmids. (b) With different DNA ratios, 12 different SRV2 retroviruses encoding GFP gene were produced in 293T cells and transduced into T cells. GFP fluorescence detected by Flow cytometry. (c-f) Retroviruses pseudotyped with original or 3 different codon optimized (CO-a, CO-b, CO-c) SRV2 envelope were produced in 293T cells. GFP genes packaged in SRV2 RVs were transduced into T cells (c, d) or NK cells (e, f). (c, e) Cell images were taken 3-5 days (T) or 7-10 days (NK) after gene transduction. Images are representative of three independent experiments with similar results. (d, f) GFP fluorescence detected by Flow cytometry was analyzed as histograms. Source data are provided as a Source Data file.

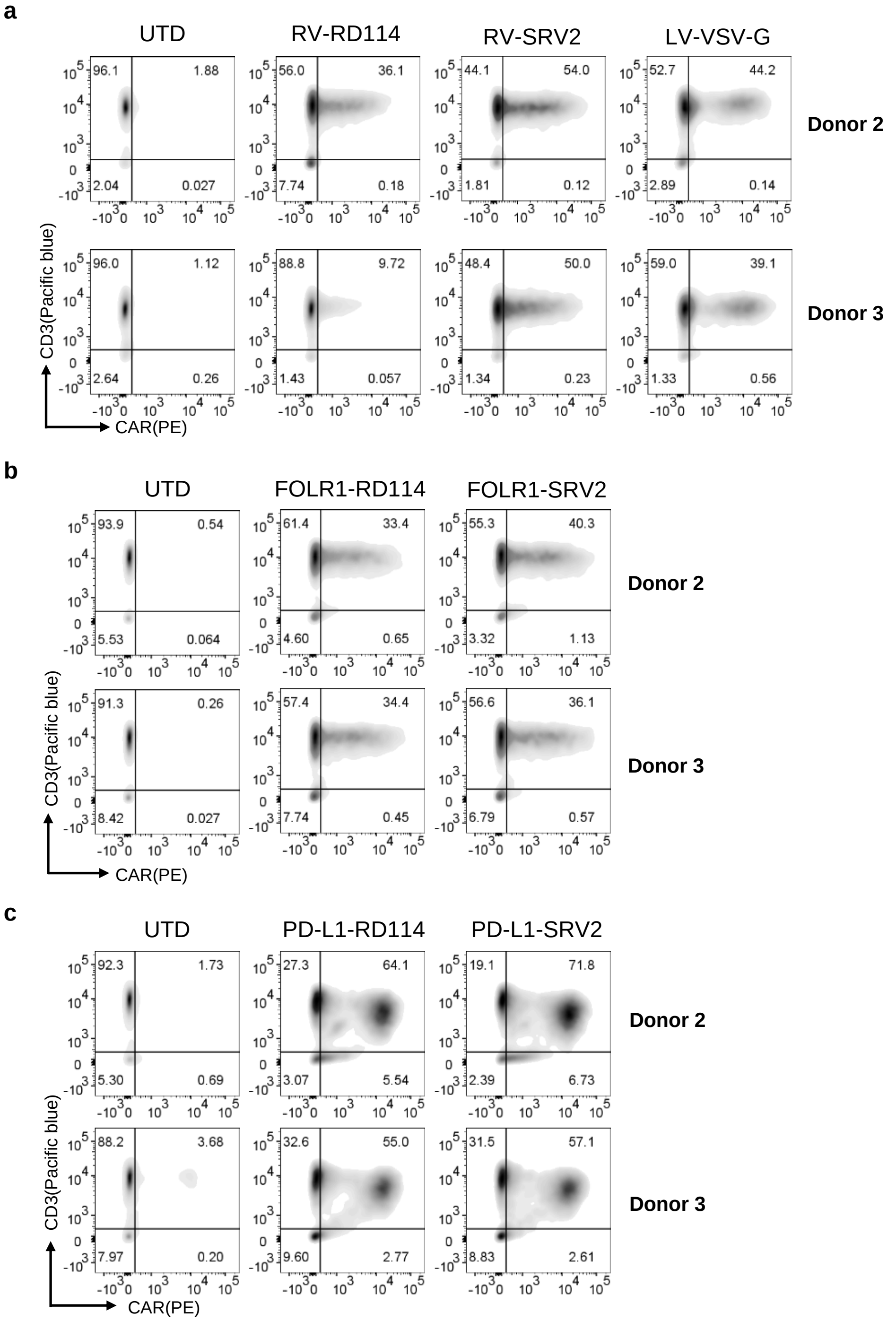

Supplementary figure 4 | CAR expression in CAR-T cells produced by RD114 RV, SRV2 RV, or VSV-G LV.

Flow cytometry analysis of CAR surface expression in CD19 (a), FOLR1 (b), and PD-L1 (c) CAR-T cells in 2 different donors. Gating strategies are provided in Supplementary fig. 7.

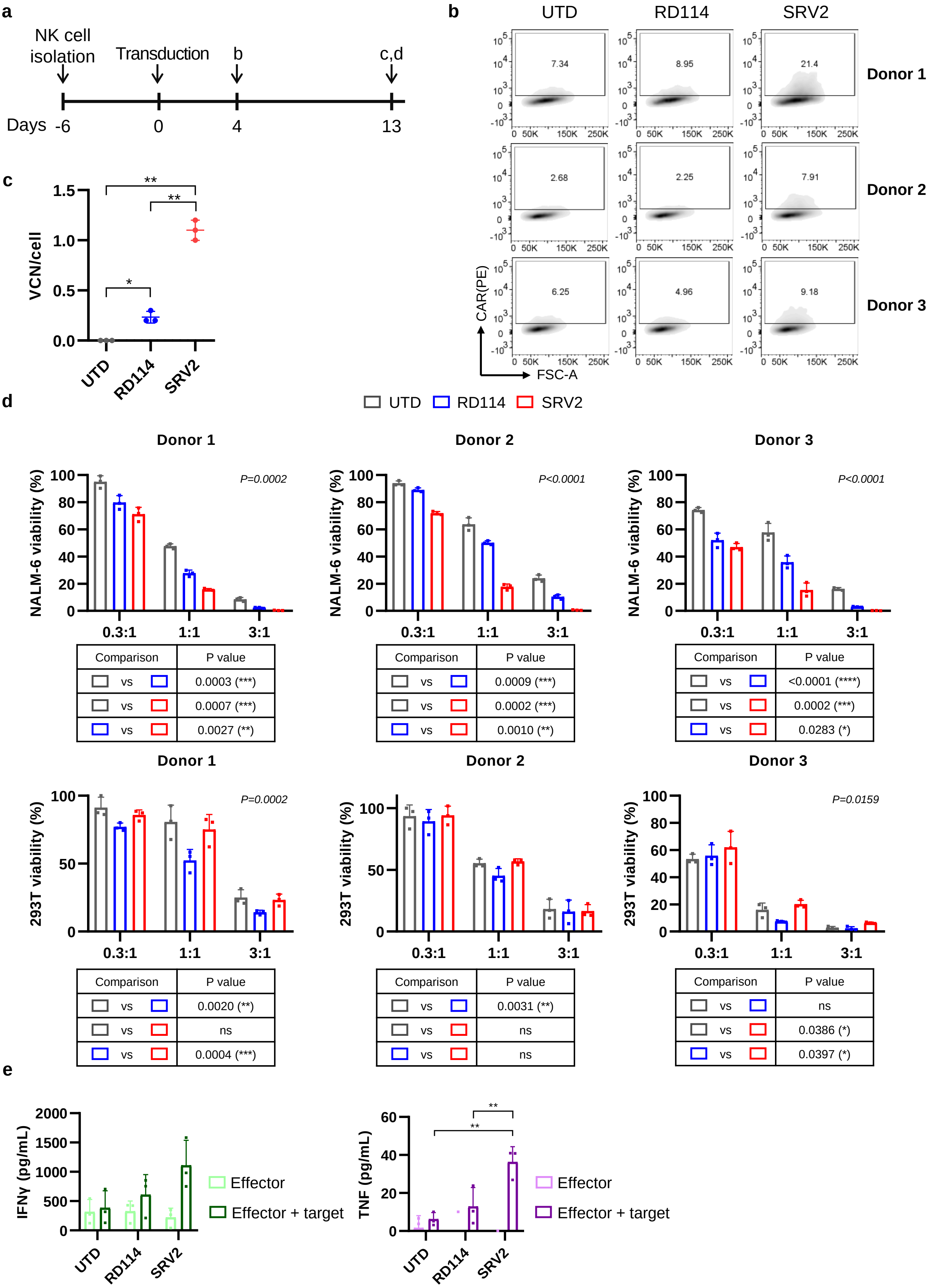
a
NK cell isolation
Transduction
b
c,d
Days -6
0
4
13
b
UTD
RD114
SRV2
Donor 1
Donor 2
Donor 3
7.34
8.95
21.4
2.68
2.25
7.91
6.25
4.96
9.18
CAR(PE)
FSC-A
c
VCN/cell
UTD
RD114
SRV2
d
UTD RD114 SRV2
Donor 1
Donor 2
Donor 3
NALM-6 viability (%)
P=0.0002
P<0.0001
P<0.0001
0.3:1
1:1
3:1
Comparison
P value
vs
0.0003 (***)
0.0007 (***)
0.0027 (**)
0.0009 (***)
0.0002 (***)
0.0010 (**)
<0.0001 (****)
0.0002 (***)
0.0283 (*)
293T viability (%)
P=0.0002
P=0.0159
0.0020 (**)
ns
0.0004 (***)
0.0031 (**)
ns
ns
ns
0.0386 (*)
0.0397 (*)
e
IFNγ (pg/mL)
Effector
Effector + target
TNF (pg/mL)
Effector
Effector + target

Supplementary figure 5 | Comparison of *in vitro* anti-cancer activity of CAR-NK cells generated by SRV2 RV or RD114 RV.

CD19 CAR-NK cells were generated from PBMCs of three independent donors using RD114 RV or SRV2 RV. (a) Schematic overview of the CAR-T cell generation and *in vitro* evaluation workflow. (b) Flow cytometry analysis of CAR surface expression in CD19 CAR-NK cells. Gating strategies are provided in Supplementary fig. 7. (c) Vector copy number (VCN) in genomic DNA, quantified by ddPCR targeting the CAR transgene and using *gapdh* (2 copies) as the reference gene. (d) Cytotoxicity of CD19 CAR-NK cells against antigen-positive NALM-6 cells and antigen-negative 293T cells after 24 hrs, measured by luminescence (n = 3 biologically independent experiments). The values 0.3, 1:1, and 3:1 in the graph represent the effector-to-target (E:T) ratios used in the assay. f. Cytokine secretion by CD19 CAR-NK cells following 24 hrs co-culture with NALM-6 cells (IFN-γ and TNF), measured by ELISA (n = 3 biologically independent experiments). Data are presented as mean ± s.d. Representative of three independent experiments. Statistical significance was determined using one-way ANOVA with Tukey's post-hoc test (e) or two-way ANOVA with Tukey's post-hoc test (f). $^{*}P < 0.05$, $^{**}P < 0.01$, $^{***}P < 0.001$, $^{****}P < 0.0001$. Source data are provided as a Source Data file.

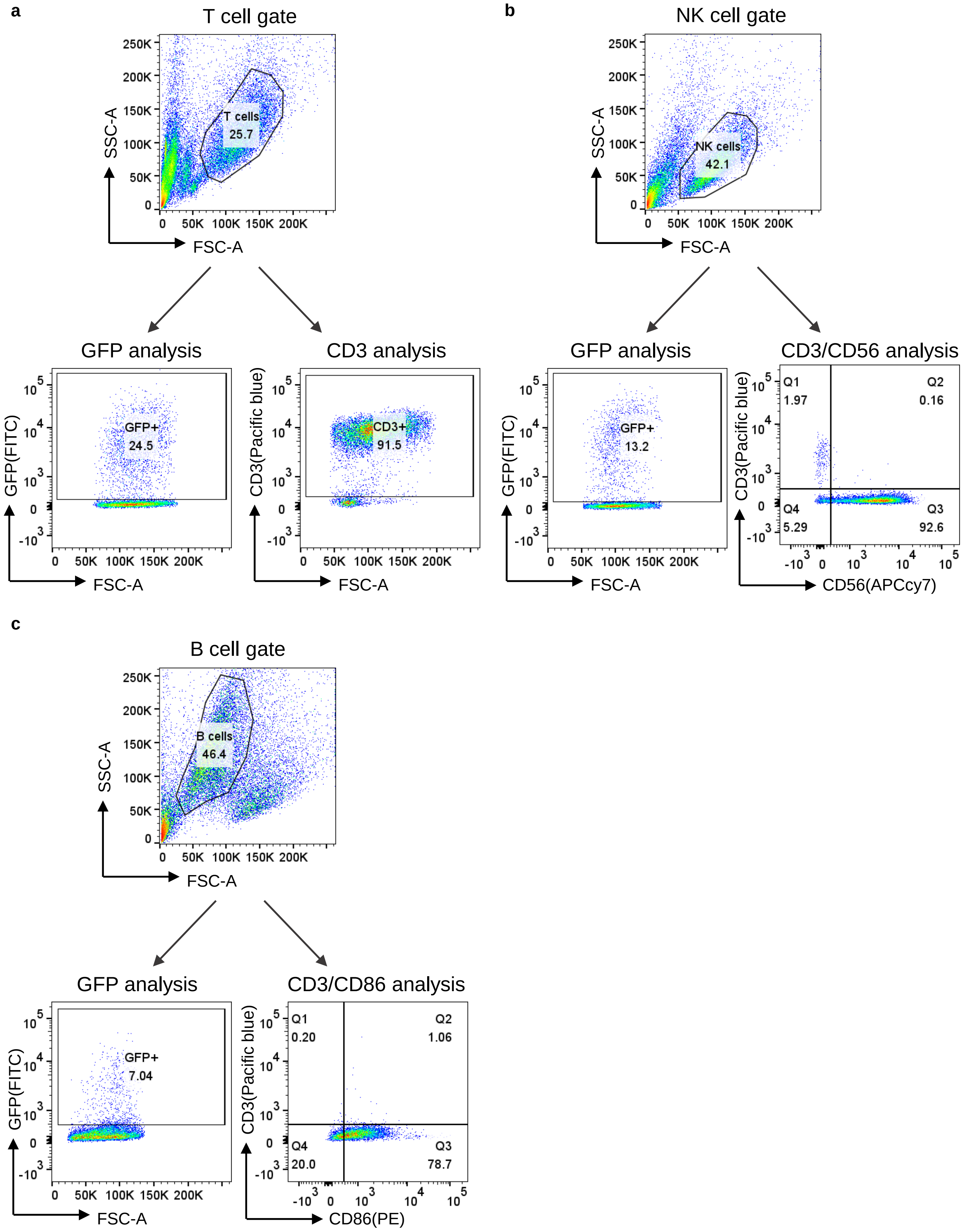

Supplementary figure 6 | Gating strategies used for cell sorting

(a) Gating strategy to sort T cells (GFP+ or CD3+) presented on Fig. 2d, 3b, 4d, Supplementary fig. 1d. (b) Gating strategy to sort NK cells (GFP+ or CD3-CD56+) presented on Fig. 2i, 3e, Supplementary fig. 1h. (c) Gating strategy to sort B cells (GFP+ or CD3-CD86+) presented on Fig. 2n, Supplementary fig. 1l.

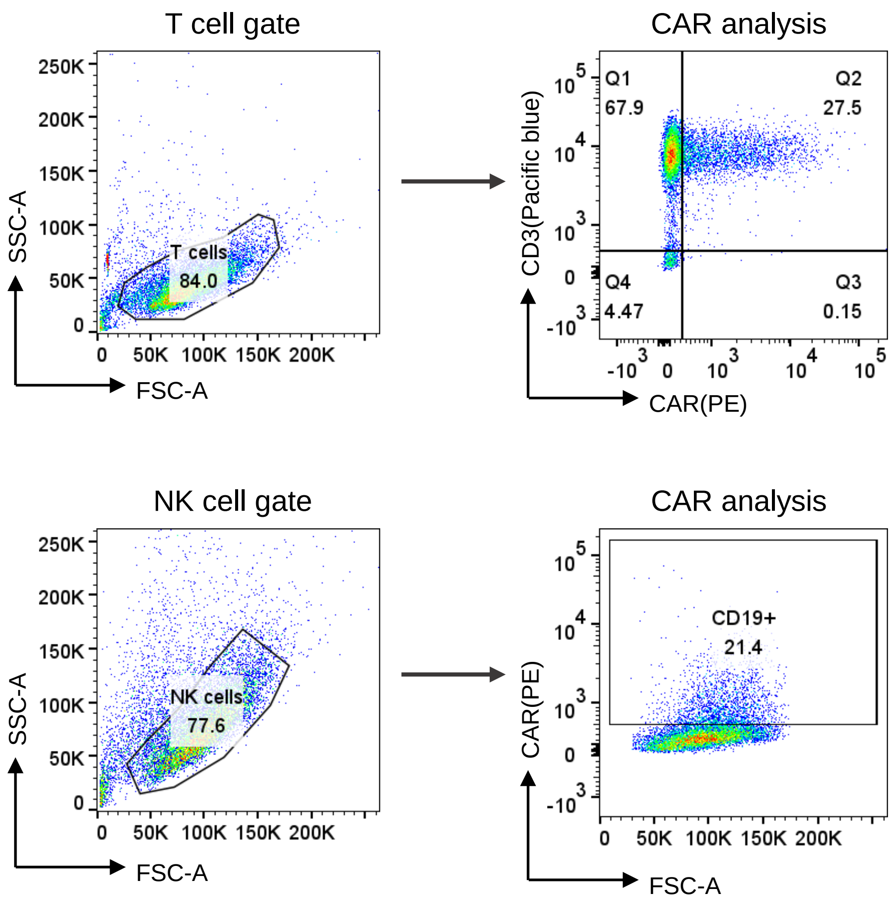

Supplementary figure 7 | Gating strategies used for cell sorting

(a) Gating strategy to sort CAR T cells (CD3+CAR+) presented on Fig. 5c, 5g, 5i, 6b, 6g, Supplementary figure 4a-c. (b) Gating strategy to sort CAR NK cells (CAR+) presented on Supplementary figure 5b.

Supplementary Table 1. Primers used in cloning

Name	Sequence(5′→3′)
SFFV_SRV2_F	ATCTAGACGCGTATGACTCTCAAGGACATC
SFFV_SRV2_R	ATCTAGACCGGTCTACGATACACGTAAATA
SRV1_F	ATCTAGACGCGTATGAACTTCAATCATCAT
SRV1_R	ATCTAGCTCGAGCTATGTTAAGTTTAAATA
SRV4_F	ATCTAGACGCGTATGAACTTCGAACACAAA
SRV4_R	ATCTAGCTCGAGTTAAGTTATCTGTAAATA
SRV5_F	ATCTAGACGCGTATGAGCAGCAAAAAAATC
SRV5_R	ATCTAGCTCGAGTTAAATGCTTAAATAGGG
SRV8_F	ATCTAGACGCGTATGAAGCTTATTCACATC
SRV8_R	ATCTAGCTCGAGCTATGTTATGCGTAAATA
CO1_F	ATCTACACGCGTATGACACTGAAGG
CO1_R	ATCTACAAGCTTCTCGAGTCAAGAGACTCTAAGGTA
CO2_F	ATCTACACGCGTATGACTCTCAAGGACATC
CO2_R	ATCTACAAGCTTCTCGAGTCAGCTCACCCGCAGATA
CO3_F	ATCTACACGCGTATGACATTGAAGGACATA
CO3_R	ATCTACAAGCTTCTCGAGCTAGCTCACTCTCAGGTA
SRV2_pro_F1	ATCTACACGCGTATGACTCTCAAGGACATC
SRV2_pro_R1	ATCTACGTCTTCCTGCTCAAGGCGCTGAGTCAAAACCAGAGCCTGGA CGGCTTGGATGGCATCTAT
SRV2_pro_F2	ATCTACATAGATGCCATCCAAGCCGTCCAGGCTCTGGTTTTGACTCAG CGCCTTGAGCAGGAAGAC
SRV2_pro_R2	ATCTACGCGGCCGCATGGGCCCAGATCTCCGA
SRV2_TR_F1	ATCTACACGCGTATGACTCTCAAGGACATC
SRV2_TR_R1	ATCTACAATCGATTGGTCCAATTTGTTAAAGACAGGATCTCAGTGGTC CAGGCTCTGGTTTTGACTCAGCAATAT
SRV2_TR_F2	ATCTACTCATGGCTCGTACTCTATGGGTTTTAGCTGGTGATATTGCTG AGTCAAAACCAGAGCCTGGACCACTGA
SRV2_TR_R2	ATCTACGCGGCCGCATGGGCCCAGATCTCCGA
SRV2_△TR_F	ATCTACACGCGTATGACTCTCAAGGACATC
SRV2_△TR_R	ATCTACACCGGTGAAAATTAAGGGTCCAAA
SRV2_△R_F	ATCTACACGCGTATGACTCTCAAGGACATC
SRV2_△R_R	ATCTACACCGGTCTGAATAGGCTTGGCTTG

Supplementary Table 2. Primers and probes used for ddPCR

Name	Sequence(5′→3′)
GFP_F	ACGACGGCAACTACAAGACC
GFP_R	GTCCTCCTTGAAGTCGATGC
CAR_F	GTGGCCGGGACCCTGAGATG
CAR_R	TGTAGGCCTCCGCCATCTTATCT
GAPDH_F	GTTGCCATCAATGACCCCTT
GAPDH_R	AAACCTGGGGGAATACGTGA
GFP_probe	(FAM)-AGGGCGACACCCTGGTGAAC-(BHQ1)
CAR_probe	(FAM)-AAGCCGAGAAGGAAGAACCCTCAGGAAGGCCT-(BHQ1)
GAPDH_probe	(HEX)-ACCTCAACTACATGGTGAGTGCTACATGGTGAG-(BHQ1)

Supplementary Table 3. Antibodies and proteins used in flow cytometry

Product	Manufacturer (Cat. No.)	Working concentration or amount used
Human CD19 (20-291) Protein, Fc Tag, premium grade	ACROBiosystems (CD9-H5251)	8 μg/mL
Human PD-L1/B7-H1 Protein, Fc Tag (MALS verified)	ACROBiosystmes (PD1-H5258)	12 μg/mL
Human FOLR1 Protein, Fc Tag (MALS verified)	ACROBiosystems (FO1-H5253)	20 μg/mL
PE anti-human IgG Fc Antibody	BioLegend (410708)	1 μL/test in 50 μL (used without additional dilution)
Pacific Blue anti-human CD3 Antibody	BioLegend (300417)	1 μL/test in 50 μL (used without additional dilution)
PE anti-human CD86 Antibody	BioLegend (305405)	1 μL/test in 50 μL (used without additional dilution)
APC/Cyanine7 anti-human CD56 (NCAM) Antibody	BioLegend (362511)	1 μL/test in 50 μL (used without additional dilution)

Supplementary Table 4. Effective MOI of GFP experiments

Experiments	Group	T	NK	B
Figure 2	RD114 RV	0.35	0.23	0.04
	SRV2 RV	0.44	0.35	0.06
Supplementary figure 1	SRV2 RV	0.41	0.31	0.06
	VSV-G LV	0.81	0.38	0.27
