# Supplementary material for: Discovery of a novel envelope protein derived from simian retrovirus 2 for pseudotyping retroviral vectors used for production of CAR immune cells
Source: Nat Commun. 2026 Apr 23;17:5643. doi: 10.1038/s41467-026-72024-4 (PMC13315792; doi:10.1038/s41467-026-72024-4)
Supplement: Supplementary file 2 — Reporting Summary [file 41467_2026_72024_MOESM2_ESM.pdf]

nature portfolio

Corresponding author(s): Chi Hoon Park
Last updated by author(s): Mar 26, 2026

Reporting Summary

Nature Portfolio wishes to improve the reproducibility of the work that we publish. This form provides structure for consistency and transparency in reporting. For further information on Nature Portfolio policies, see our Editorial Policies and the Editorial Policy Checklist.

Statistics

For all statistical analyses, confirm that the following items are present in the figure legend, table legend, main text, or Methods section.

n/a	Confirmed	
☐	☒	The exact sample size (*n*) for each experimental group/condition, given as a discrete number and unit of measurement
☐	☒	A statement on whether measurements were taken from distinct samples or whether the same sample was measured repeatedly
☐	☒	The statistical test(s) used AND whether they are one- or two-sided<br>*Only common tests should be described solely by name; describe more complex techniques in the Methods section.*
☒	☐	A description of all covariates tested
☐	☒	A description of any assumptions or corrections, such as tests of normality and adjustment for multiple comparisons
☐	☒	A full description of the statistical parameters including central tendency (e.g. means) or other basic estimates (e.g. regression coefficient) AND variation (e.g. standard deviation) or associated estimates of uncertainty (e.g. confidence intervals)
☐	☒	For null hypothesis testing, the test statistic (e.g. *F*, *t*, *r*) with confidence intervals, effect sizes, degrees of freedom and *P* value noted<br>*Give P values as exact values whenever suitable.*
☒	☐	For Bayesian analysis, information on the choice of priors and Markov chain Monte Carlo settings
☒	☐	For hierarchical and complex designs, identification of the appropriate level for tests and full reporting of outcomes
☒	☐	Estimates of effect sizes (e.g. Cohen's *d*, Pearson's *r*), indicating how they were calculated

Our web collection on statistics for biologists contains articles on many of the points above.

Software and code

Policy information about availability of computer code

Data collection	-
Data analysis	-

For manuscripts utilizing custom algorithms or software that are central to the research but not yet described in published literature, software must be made available to editors and reviewers. We strongly encourage code deposition in a community repository (e.g. GitHub). See the Nature Portfolio guidelines for submitting code & software for further information.

Data

Policy information about availability of data

All manuscripts must include a data availability statement. This statement should provide the following information, where applicable:

- Accession codes, unique identifiers, or web links for publicly available datasets
- A description of any restrictions on data availability
- For clinical datasets or third party data, please ensure that the statement adheres to our policy

All data are included in the Supplementary Information or available from the authors, as are unique reagents used in this Article. The raw numbers for charts and graphs are available in the Source Data file whenever possible.

Research involving human participants, their data, or biological material

Policy information about studies with human participants or human data. See also policy information about sex, gender (identity/presentation), and sexual orientation and race, ethnicity and racism.

Reporting on sex and gender	Sex/gender information was not available for the healthy donors whose blood samples were used in this study. Therefore, sex/gender was not considered in the study design or analysis.
Reporting on race, ethnicity, or other socially relevant groupings	Race/ethnicity data were not collected.
Population characteristics	Peripheral blood samples were obtained from healthy adult human donors for isolation of PBMCs, as applicable.
Recruitment	Healthy donor blood samples were obtained through Daejeon-Sejong-Chungnam Blood Center under an IRB approved protocol.
Ethics oversight	The use of blood samples from healthy human donors was approved by the Institutional Review Board of the Korea National Institute for Bioethics Policy (Approval no. P01-201607-31-003). Written informed consent was obtained from all donors prior to sample collection.

Note that full information on the approval of the study protocol must also be provided in the manuscript.

Field-specific reporting

Please select the one below that is the best fit for your research. If you are not sure, read the appropriate sections before making your selection.

- [x] Life sciences
- [ ] Behavioural & social sciences
- [ ] Ecological, evolutionary & environmental sciences

For a reference copy of the document with all sections, see nature.com/documents/nr-reporting-summary-flat.pdf

Life sciences study design

All studies must disclose on these points even when the disclosure is negative.

Sample size	For in vitro experiments, each data point represents an independent biological replicate derived from separate viral preparations and/or independent donor-derived primary cells, with a minimum of three biological replicates per condition. For experiments involving primary human T, NK, or B cells, cells from multiple independent donors were used, and donor numbers are indicated in the figure legends.<br>For in vivo experiments, group sizes were chosen based on previous studies using similar animal models and endpoints(n=4), and all animals meeting predefined inclusion criteria were included in the analysis.
Data exclusions	There are no data were excluded from the analyses.
Replication	All relevant data from biological replicates are shown in the manuscript. Experiments were repeated with the same conditions and obtained similar results.
Randomization	All samples were randomized to ensure no bias was introduced by investigators.
Blinding	Experimental data were gathered in a non-blinded fashion. Data analysis was performed without a prior knowledge of the experimental design.

Reporting for specific materials, systems and methods

We require information from authors about some types of materials, experimental systems and methods used in many studies. Here, indicate whether each material, system or method listed is relevant to your study. If you are not sure if a list item applies to your research, read the appropriate section before selecting a response.

Materials & experimental systems

n/a	Involved in the study	
[ ]	[x]	Antibodies
[ ]	[x]	Eukaryotic cell lines
[x]	[ ]	Palaeontology and archaeology
[ ]	[x]	Animals and other organisms
[x]	[ ]	Clinical data
[x]	[ ]	Dual use research of concern
[x]	[ ]	Plants

Methods

n/a	Involved in the study	
[x]	[ ]	ChIP-seq
[ ]	[x]	Flow cytometry
[x]	[ ]	MRI-based neuroimaging

Antibodies

Antibodies used	Flow cytometry : Human CD19(20-291) Proteins, Fc Tag (ACROBiosystems, CD9-H5251), Human PD-L1/B7-H1 Protein, Fc Tag (ACROBiosystems, PD1-H5258), Human FOLR1 Protein, Fc Tag (ACROBiosytems, FO1-H5253), PE anti-human IgG Fc Antibody (BioLegend, 410708), Pacific Blue anti-human CD3 Antibody (BioLegend, 300417), FITC anti-human CD19 Antibody (BioLegend, 363007), PE anti-human CD86 Antibody (Biolegend, 305405), and APC/Cyanine7 anti-human CD56(NCAM) Antibody (BioLegend, 362511)
Validation	All commercial antibodies were validated by their suppliers: ACROBiosystems and BioLegend.

Eukaryotic cell lines

Policy information about cell lines and Sex and Gender in Research

Cell line source(s)	293T, NALM-6, A549, HeLa, HepG2, and HCT-8 cells were purchased from ATCC. NUGC-3 cell was purchased from JCRB.
Authentication	Each cell line was handled and cultured separately, stored at early passages, and discarded after more than 20 passages. All these measures helped to preserve cell identity.
Mycoplasma contamination	All cell lines were confirmed negative for mycoplasma.
Commonly misidentified lines (See ICLAC register)	No commonly misidentified cell lines were used.

Animals and other research organisms

Policy information about studies involving animals; ARRIVE guidelines recommended for reporting animal research, and Sex and Gender in Research

Laboratory animals	Six- to eight-week-old female NOD-SCID IL2R γ null (NSG) mice were obtained from Charles River Laboratories Japan, Inc. All procedures are approved by the Laboratory Animal Care and Use Committee of the Korea Research Institute of Chemical Technology and were conducted in accordance with the Institute for Laboratory Animal Research Guide for the Care and Use of Laboratory Animals. All efforts were made to minimize animal suffering.
Wild animals	This study did not involve wild animals.
Reporting on sex	Female mice that do not have a tendency to fight were used in this experiment.
Field-collected samples	This study did not involve field-collected samples.
Ethics oversight	The animal experiments were approved by the Laboratory Animal Care and Use Committee of the Korea Research Institute of Chemical Technology.

Note that full information on the approval of the study protocol must also be provided in the manuscript.

Plants

Seed stocks	-
Novel plant genotypes	-
Authentication	-

Flow Cytometry

Plots

Confirm that:

- [x] The axis labels state the marker and fluorochrome used (e.g. CD4-FITC).
- [x] The axis scales are clearly visible. Include numbers along axes only for bottom left plot of group (a 'group' is an analysis of identical markers).
- [x] All plots are contour plots with outliers or pseudocolor plots.
- [x] A numerical value for number of cells or percentage (with statistics) is provided.

Methodology

Sample preparation	Control or experimental cells were placed in 1.5 mL EP tubes and centrifuged at 4,000 rpm for 5 minutes at 4°C. After removing the supernatant, the cells were washed twice with staining buffer (PBS containing 0.2% BSA and 0.08% NaN3). The cells were then resuspended in 50 μL of staining buffer, and 1 μL of antibody or protein was added for primary staining, which was performed for 30 minutes. For secondary staining, if needed, the cells were washed twice and stained using the same procedure as for primary staining. After two additional washes, the cells were fixed with 250 μL of fixation buffer (PBS containing 0.4% paraformaldehyde) for 10 minutes, followed by two more washes and filtration through a cell strainer. For GFP expressing cells, the same centrifugation and washing steps were applied, followed by fixation with fixation buffer, two additional washes, and filtration through a cell strainer.
Instrument	Cells were analyzed using BD FACS canto Ⅱ.
Software	FACS data were collected using BD FACSDiva (BD Biosciences), and data analysis was performed using Flowjo (version 10.10.0).
Cell population abundance	Abundance of cell populations within flow-cytometry results are specified throughout the manuscript in reference to specific experiments.
Gating strategy	Cells were gated on SSC-A vs. FSC-A, selecting the major cell population in accordance with published datasets.

- [x] Tick this box to confirm that a figure exemplifying the gating strategy is provided in the Supplementary Information.
