# Supplementary material for: Discovery of a novel envelope protein derived from simian retrovirus 2 for pseudotyping retroviral vectors used for production of CAR immune cells
Source: Nat Commun. 2026 Apr 23;17:5643. doi: 10.1038/s41467-026-72024-4 (PMC13315792; doi:10.1038/s41467-026-72024-4)
Supplement: Supplementary file 3 — Transparent Peer Review file [file 41467_2026_72024_MOESM3_ESM.pdf]

nature portfolio

Peer Review File

Discovery of a novel envelope protein derived from simian retrovirus 2 for pseudotyping retroviral vectors used for production of CAR immune cells

Corresponding Author: Dr Chi Hoon Park

This file contains all reviewer reports in order by version, followed by all author rebuttals in order by version.

Version 0:

Reviewer comments:

Reviewer #1

(Remarks to the Author)
This article explores the construction of pseudovirus vectors using SRV-2 envelope protein for the generation of CAR-T and CAR-NK cells with potential for immunotherapy. By comparing with the widely used RD114 pseudovirus vectors, the study shows that SRV-2 pseudoviruses demonstrate significantly higher gene transduction efficiency in T cells and NK cells than RD114. The efficacy of SRV-2 pseudovirus vectors in tumor immunotherapy was also validated both in vitro and in vivo.

Major:
1.The Materials and Methods section can be further detailed. For instance, the optimization process of SRV-2, along with the specific steps and conditions for its transduction in CAR-T and CAR-NK cells and cell culture, can be described in greater detail.
2.Figure 1 lacks a negative control experiment, such as using an untransduced (UTD) group, to further confirm whether the observed differences in transduction efficiency are due to the virus itself.
3.In Figures 1 and 2, were the experiments repeated multiple times? The results should reflect the standard deviation or standard error of each group to ensure the reproducibility and statistical significance of the results.
4.Figure 2 might consider involving cells from different sources in the experiment to assess the universality of SRV-2 transduction.
5.For the results of Figure 3, the article does not discuss why other SRV subtypes failed to effectively transduce genes. There might be an impact of surface glycoprotein sequence differences between SRV subtypes, but the article lacks an in-depth molecular mechanism analysis.
6.Figure 4 describes an attempt to use SRV-2 envelope protein in lentiviral vectors, but no effective gene transduction was achieved. This might involve an incompatibility between lentiviral vectors and SRV-2 envelope protein in the membrane fusion mechanism, and a discussion of the related mechanism should be added.
7.In the optimization of production in Figure 5, was the balance between cytotoxicity, yield, and transduction efficiency considered? Detailed scientific explanations should be provided for the selection of each adjusted parameter, along with the specific impact of these parameters on the results.
8.The experiments in Figures 6 & 7 show that CAR-T cells generated by SRV-2 exhibit stronger cytotoxicity in vitro. Further investigation into the killing mechanisms of different effector cells is necessary. For example, whether specific cytokines or surface marker changes are involved, and whether there is a synergistic effect of cytokines.
9.In Figure 8, the mouse experiment includes only 4 mice, which may not be statistically sufficient to draw meaningful conclusions. It is recommended to increase the sample size for more precise comparisons of survival rates and tumor volume.
10.In Figure 8, although CAR-T cells show a delay in tumor growth, the tumor size was monitored only for 60 days. Such experiments should consider longer observation periods to ensure long-term antitumor effects, especially in cases where immune system recovery is considered.
11.The article does not fully discuss the potential limitations of the experimental design, such as the variation in transduction efficiency across different cell lines and challenges encountered in optimizing viral vector production and transduction efficiency.
Minor:
1.In the Introduction, "Pseudotyped retroviruses or lentiviruses," it is recommended to change "or" to "and".

2.In Materials and Methods, the phrase "envelope proteins" lacks the article "the" before it.
3.In the Discussion, "For robust production of viral vectors, it is important for envelope proteins not to induce toxicity in virus producing cells." It is suggested to change this to "...it is important that...".
4.In the Materials and Methods section, "T cells were used for experiments within 1 to 3 days after activation." It is recommended to modify it to: "T cells were used in experiments 1 to 3 days after activation."In the Introduction or Discussion, it may be worth considering comparisons with other types of viral vectors (such as AAV vectors) in terms of transduction efficiency, toxicity, capacity, etc., to enhance the significance of the article. Relevant references, such as PMID: 38307819, PMID: 38783157, etc., could be referred to and cited.

Reviewer #2

(Remarks to the Author)
In this manuscript, Jeun et al describe a novel viral pseudotyping strategy for retroviral vectors. They use the envelope protein from Simian Retrovirus 2 to pseudotype gammaretroviral vectors commonly used in gene delivery for CAR-T cells and other applications. They show that the SRV2 vectors can effectively transduce immune cells, and that they produce CAR-T and CAR-NK cells that are functional and potentially modestly improved from RD114-pseudotyped vectors.

While there is interest in examining additional viral pseudotyping approaches, and the work here is fairly convincing that SRV2 envelope protein can be used on retroviral vectors, a lack of replication and quantitation in this paper significantly limits enthusiasm for this work.

1) While the authors mention titering the viruses in the methods section, I don’t see where that is used in the manuscript. It is thus very hard to understand (for example) if the differences observed in Figure 2C for T cells is due to an improved transduction rate for SRV2, or what could be as little as a 2-fold difference in viral titers in the experiment (which is typically well within stochastic experimental variation). The authors should provide (a) the viral titers upon production of the vector as measured via p24 and/or viral genomes, (b) the MOI for the infection assay, and (c) ideally, transductions conducted at multiple MOI to show exactly how different the SRV2 and RD114 viruses are from each other.
2) The paper would be significantly improved by better quantification and replication everywhere – multiple batches of virus with reported titers (to allow statistics), multiple transduction experiments to show how transduction rates vary.
3) Similarly, it would be necessary to show quantified percentages of cells that are transduced (for example, in Figure 2C, G, K) rather than just the histograms to help understand the differences being shown.
4) It is curious the authors do not provide VSVG pseudotyped viruses as comparison given they are by far the predominantly used gene transfer technology. To maximize the benefit of the work, the authors should include this comparison at matched p24.
5) The fact that only SRV2 works, and it only works on retroviruses rather than lentiviruses, are both interesting – can the authors provide more hypotheses for why this may be the case?
6) The optimizations in Figure 5 would likely be ok in a supplement.
7) For the functional data in figure 6, can the authors (a) show the E:T ratio for the experiment, (b) explain why there is such a viability loss in NALM6 when cocultured with untransduced T cells, and (c) explain the seeming CAR-dependent loss in cell viability for HEK cells (which are CD19-)?
8) Figure 6B – can the authors speculate what is driving higher CAR expression, given the transfer vector is the same? Perhaps perform a VCN assay? The most likely explanation seems like it may be that some of the cells may be multiply transduced, which itself could be affecting function of the CAR-T cells (which is a second order effect of the pseudotyping strategy).
9) Figure 6D – I worry that many of these changes are minor enough that they would not be functionally relevant even if they end up statistically significant.
10) Functional data in general would be more convincing if shown in at least 1-2 more donors.
11) Figure 7- can the authors explain a bit more what the difference is between RV and RV2? Comparing RD114 and RV2-SRV2 feels like an unfair comparison given how much effect the change in transfer vector is clearly having.
12) Figure 8 – this demonstrates that the CAR is functional, but it completely drops the comparison to any other approaches, limiting its demonstration of utility compared to RD114-Retro, VSV-retro, or (the by far most commonly used) VSV-lenti.

Reviewer #3

(Remarks to the Author)
The manuscript by Jeun and colleagues explored a glycoprotein derived from simian retrovirus 2 for pseudotyping of retroviruses. Initially the authors established that of the eight serotypes of SRV the glycoprotein of serotype 2 seems to be the most potent one for transduction of T- and NK cells. Next, the authors produced respective pseudotyped retroviruses. However, production of pseudotyped lentiviral vectors was not feasible. After optimizing the protocol for production of SRV2 pseudotyped retroviral vectors the authors produced vectors encoding the CD19 CAR and demonstrate that this is advantageous compared to RD114 pseudotyped retroviruses. As a final experiment, the authors show antitumor effects in vivo after administration of the produced CAR-T cells using the SRV2 pseuotyped vector.
This is an interesting study. However, I am not sure whether the advancement in the field is big enough to argue publication in this journal. Moreover, some important information is lacking as specified in the comments below.

Major comments:
• The authors state that the SRV2 RV exhibited approximately 20% more CAR-positive T cells and 7% more CAR-positive NK cells compared to RD114 pseudotyped vectors, respectively. In general, I am not entirely sure whether this advancement

especially in NK cells is big enough. In contrast, the data for the modified transfer vector RV2 seem very promising in NK cells. Maybe I missed that information but I think the authors should specify for figure 7B what exactly the modified transfer vector (RV2) looks like. This information should be added to the materials and methods or results section and the phenomenon should be discussed.
• In the discussion section the authors claim the results of this manuscript show that SRV-2 pseudotyped retroviruses outperform RD114 pseudotyped retroviruses both in vitro and in vivo. However, there are no results displayed for the RD114 virus in vivo in Figure 8. The authors should perform these experiments.
• Is it possible to also test the CAR-NK cells in vivo? Have the authors performed these experiments?

Minor comments:
• Figures 2: For subfigures D, H, L it should be stated in the figure legend how many biological and technical replicates were performed? It would be advantageous to add some statistics to these subfigures. The authors display fluorescent intensity for the different groups. How about the percentage of cells, which were transduced?
• Figure 3: For subfigures C and F, it should be stated in the figure legend how many biological and technical replicates were performed? As already stated in the previous comment the percentage of cells which were transduced should be mentioned (not only the fluorescent intensity)?
• Whenever suitable in Figures 2 and 3 statistics should be added.

Version 1:

Reviewer comments:

Reviewer #1

(Remarks to the Author)
The authors have made extensive and thoughtful revisions to address the majority of concerns raised in the initial review, and the revised manuscript is now substantially improved. This work now provides a well-supported evaluation of SRV2-pseudotyped retroviral vectors for CAR-T/NK engineering, with clear comparative data against the gold-standard RD114 envelope. My further suggestions are as follows:

1.The authors has added VCN data to link transgene copy number with CAR expression and cytotoxicity, a key response to initial requests for mechanistic validation of functional improvements. However, a critical translational limitation remains unaddressed: the study does not examine integration-site preferences or potential genotoxic risk of SRV2-pseudotyped retroviruses. Retroviral insertion near proto-oncogenes is a well-documented cause of leukemogenesis in gene therapy trials (PMID: 37175441), and safety-efficacy balance is central to the clinical potential of this vector system. While comprehensive integration profiling may be beyond the present scope, this limitation must be explicitly acknowledged and discussed in detail in the Discussion section, with a clear note on how this gap impacts the translational interpretability of the study's findings.
2.The addition of VCN data raises a critical new interpretative point that the authors must address explicitly: the functional advantage of SRV2-pseudotyped vectors appears to be quantitative rather than qualitative. That is, improved efficacy stems from increased transgene copies per cell, not a unique biological property of SRV2 itself. The authors must explicitly clarify and discuss this distinction in the manuscript to avoid overstating mechanistic novelty. For context, Norberg et al. (PMID:41445185) quantified this exact VCN-dependent effect on CAR-T function, and this reference should be integrated to frame the study's findings appropriately.
3.Mechanism experiments still lack effective supplementation. However, I think the author's reply is reasonable.
4.Extension of the in vivo follow-up substantially strengthens the study. Nonetheless, the relatively small cohort size should be more explicitly acknowledged as a limitation in the Discussion.
5.Infection parameters and normalization strategies should be more clearly described in the Methods to facilitate reproducibility. For example, incubation time, incubation temperature, and the volume ratio of viral solution to cell suspension.
6.Ensure consistent terminology when referring to vector types and pseudotyping strategies throughout the manuscript.
7.Please ensure that sample sizes, statistical tests, and definitions of error bars are consistently reported across all figure legends.

Reviewer #2

(Remarks to the Author)
While I still think many of the differences observed here are quite subtle, the paper is strengthened and there will be interest in continued expansion of alternative pseudo typing strategies- so I think I is reasonable to publish in this form.

Reviewer #3

(Remarks to the Author)
The authors addressed all raised issues and performed additional experiments to address ramaining points and to add respective statistical analyses. Therefore, acceptance of this manuscript can be recommended.

Open Access This Peer Review File is licensed under a Creative Commons Attribution 4.0 International License, which permits use, sharing, adaptation, distribution and reproduction in any medium or format, as long as you give appropriate credit to the original author(s) and the source, provide a link to the Creative Commons license, and indicate if changes were made.
In cases where reviewers are anonymous, credit should be given to 'Anonymous Referee' and the source.
The images or other third party material in this Peer Review File are included in the article’s Creative Commons license, unless indicated otherwise in a credit line to the material. If material is not included in the article’s Creative Commons license and your intended use is not permitted by statutory regulation or exceeds the permitted use, you will need to obtain permission directly from the copyright holder.
To view a copy of this license, visit https://creativecommons.org/licenses/by/4.0/

Reviewer #1 (Remarks to the Author):

This article explores the construction of pseudovirus vectors using SRV-2 envelope protein for the generation of CAR-T and CAR-NK cells with potential for immunotherapy. By comparing with the widely used RD114 pseudovirus vectors, the study shows that SRV-2 pseudoviruses demonstrate significantly higher gene transduction efficiency in T cells and NK cells than RD114. The efficacy of SRV-2 pseudovirus vectors in tumor immunotherapy was also validated both in vitro and in vivo.

Major:
1.The Materials and Methods section can be further detailed. For instance, the optimization process of SRV-2, along with the specific steps and conditions for its transduction in CAR-T and CAR-NK cells and cell culture, can be described in greater detail.

→ We thank the reviewer for this helpful suggestion. The SRV2 RV production process used for optimization follows the same pseudovirus production procedure described in Section 3 of Methods ("Production and titration of pseudotyped lentiviruses or retroviruses"), with the only differences being the adjustment of DNA ratios, as shown in Supplementary Fig. 3a, and the use of a codon-optimized SRV2 envelope plasmid in certain experiments. In addition, the ratios of cells to viral particles used for generating CAR-T and CAR-NK cells are now clearly described in Section 6 ("Viral vector-mediated cell transduction").

2.Figure 1 lacks a negative control experiment, such as using an untransduced (UTD) group, to further confirm whether the observed differences in transduction efficiency are due to the virus itself.

→ As pointed out by the reviewer, the original Figure 1 lacked an adequate negative control. In the revised figure (Fig. 1c), we added an untransfected (UTD) group as a negative control, and we also included the RV-VSV-G and LV-VSV-G groups used for pseudovirus production. These additions make the comparison of overall transduction efficiency clearer.

3.In Figures 1 and 2, were the experiments repeated multiple times? The results should reflect the standard deviation or standard error of each group to ensure the reproducibility and statistical significance of the results.

→ We agree that reproducibility is highly important. Although the original Figure 1 did not include quantified graphs, repeated experiments using T, NK, and B cells generated from PBMCs of multiple donors were performed for Figure 2, and these results were incorporated. Standard deviations and statistical analyses based on repeated experiments have also been added.

4.Figure 2 might consider involving cells from different sources in the experiment to assess the universality of SRV-2 transduction.

→ In accordance with the reviewer's suggestion, we conducted repeated experiments using T, NK, and B cells activated from PBMCs derived from different donors. Consistent SRV2-mediated gene-delivery patterns were observed across donors, supporting the universality of SRV2-based transduction.

5.For the results of Figure 3, the article does not discuss why other SRV subtypes failed to effectively transduce genes. There might be an impact of surface glycoprotein sequence differences between SRV subtypes, but the article lacks an in-depth molecular mechanism analysis.

→ We appreciate this important point. To address it, we performed additional experiments using diverse tissue-derived cell types to evaluate whether SRV serotypes exhibit tissue-specific differences. Although clear tissue specificity was not observed, SRV2 consistently showed higher infectivity across all cell types tested. This suggests that differences in envelope protein sequences among SRV serotypes can contribute to differences in viral entry efficiency independently of tissue origin. These mechanistic interpretations have been added to the revised Discussion. While advanced structural or binding-prediction analyses are beyond the scope of the present work, the new experimental

findings and supporting literature collectively address the reviewer's concern and strengthen our interpretation.

6.Figure 4 describes an attempt to use SRV-2 envelope protein in lentiviral vectors, but no effective gene transduction was achieved. This might involve an incompatibility between lentiviral vectors and SRV-2 envelope protein in the membrane fusion mechanism, and a discussion of the related mechanism should be added.

→ We thank the reviewer for the valuable comment. Previous literature indicates that the cytoplasmic domain (CT) of SRV2 is likely not efficiently cleaved by the lentiviral protease, resulting in inadequate incorporation of the envelope protein into lentiviral particles. We attempted multiple CT modifications; although some variants exhibited modest improvements in transduction efficiency, they remained substantially lower than VSV-G pseudotyping. Thomas et al. (2019) also reported that substituting the RD114 CT with the 4070A CT yields lower titers than replacing its protease-cleavage motif with an HIV-1 sequence, yet neither reaches the high titers achieved with VSV-G. Collectively, these findings and our experimental data indicate that the fundamental limitation lies in low MA–CT interaction efficiency in the lentiviral system. We have incorporated this mechanistic explanation into the Discussion.

7.In the optimization of production in Figure 5, was the balance between cytotoxicity, yield, and transduction efficiency considered? Detailed scientific explanations should be provided for the selection of each adjusted parameter, along with the specific impact of these parameters on the results.

→ As the reviewer noted, we have added detailed scientific rationale for each optimized parameter in the Results section ("Establishing the optimized protocol for producing SRV2 RV"). We also describe how each condition influences viral yield, cytotoxicity, and transduction efficiency, thereby clarifying the logic behind establishing the optimized protocol.

8.The experiments in Figures 6 & 7 show that CAR-T cells generated by SRV-2 exhibit stronger cytotoxicity in vitro. Further investigation into the killing mechanisms of different effector cells is necessary. For example, whether specific cytokines or surface marker changes are involved, and whether there is a synergistic effect of cytokines.

→ We thank the reviewer for this insightful comment. Although mechanistic studies involving specific cytokines or surface-marker changes would be meaningful, such analyses fall outside the primary scope of this work, which focuses on comparing SRV2 RV and RD114 RV in terms of gene-delivery efficiency. To evaluate whether the enhanced cytotoxicity of SRV2 CAR-T cells may result from increased CAR expression, we performed a vector copy number (VCN) assay (Fig. 5d). These results support the interpretation that SRV2 RV enables more efficient gene transfer.

9.In Figure 8, the mouse experiment includes only 4 mice, which may not be statistically sufficient to draw meaningful conclusions. It is recommended to increase the sample size for more precise comparisons of survival rates and tumor volume.

→ We acknowledge this limitation. Although we were unable to repeat the same experimental conditions with a larger cohort, we performed an additional in vivo experiment directly comparing RD114 RV and SRV2 RV derived CAR-T cells (Fig. 6f–j). This additional dataset further strengthens our conclusions.

10.In Figure 8, although CAR-T cells show a delay in tumor growth, the tumor size was monitored only for 60 days. Such experiments should consider longer observation periods to ensure long-term antitumor effects, especially in cases where immune system recovery is considered.

→ As suggested by the reviewer, we extended the monitoring period to 150 days in the additional in vivo experiment comparing RD114 CAR-T and SRV2 CAR-T cells. Mice that did not show early tumor regrowth remained tumor-free until the end of the experiment. This updated information has been incorporated into the revised manuscript.

11.The article does not fully discuss the potential limitations of the experimental design, such as the variation in transduction efficiency across different cell lines and challenges encountered in optimizing viral vector production and transduction efficiency.

→ We thank the reviewer for this important comment. As the reviewer noted, transduction efficiency varies across T, NK, and B cells. To account for these intrinsic differences, we adjusted the ratio of viral particles (based on physical titers) for each cell type and transfer vector when performing the experiments. These details are described in Methods section 6, "Viral vector–mediated cell transduction." In addition, we used PBMCs obtained from multiple donors to ensure that the observed transduction efficiencies were not donor-specific but rather represented consistent patterns across biological replicates. We also expanded the Results section to address technical challenges in viral-vector production. Because our goal was to directly compare SRV2 RV and RD114 RV under identical conditions, all experiments were conducted using equal total physical-particle numbers, minimizing variability and enhancing the reliability of the comparison.

Minor:
1.In the Introduction, "Pseudotyped retroviruses or lentiviruses," it is recommended to change "or" to "and".

→ As suggested by the reviewer, the phrase has been revised to "pseudotyped retroviruses and lentiviruses." We appreciate the helpful correction.

2.In Materials and Methods, the phrase "envelope proteins" lacks the article "the" before it.

→ We revised the expression to "the envelope proteins" as suggested.

3.In the Discussion, "For robust production of viral vectors, it is important for envelope proteins not to induce toxicity in virus producing cells." It is suggested to change this to "...it is important that...".

→ To improve clarity, we have revised the sentence structure.

4.In the Materials and Methods section, "T cells were used for experiments within 1 to 3 days after activation." It is recommended to modify it to: "T cells were used in experiments 1 to 3 days after activation."In the Introduction or Discussion, it may be worth considering comparisons with other types of viral vectors (such as AAV vectors) in terms of transduction efficiency, toxicity, capacity, etc., to enhance the significance of the article. Relevant references, such as PMID: 38307819, PMID: 38783157, etc., could be referred to and cited.

→ We reviewed the suggested references (PMID: 38307819, 38783157). While the comparison is valuable, broad evaluation of other vector systems such as AAV falls outside the scope of this study, which focuses on establishing SRV2-based retroviral pseudotyping and directly comparing SRV2 RV with RD114 RV. Therefore, the suggested comparison was not included, although the cited literature contributed to broader contextual understanding.

Reviewer #2 (Remarks to the Author):

In this manuscript, Jeun et al describe a novel viral pseudotyping strategy for retroviral vectors. They use the envelope protein from Simian Retrovirus 2 to pseudotype gammaretroviral vectors commonly used in gene delivery for CAR-T cells and other applications. They show that the SRV2 vectors can effectively transduce immune cells, and that they produce CAR-T and CAR-NK cells that are functional and potentially modestly improved from RD114-pseudotyped vectors.

While there is interest in examining additional viral pseudotyping approaches, and the work here is fairly convincing that SRV2 envelope protein can be used on retroviral vectors, a lack of replication and quantitation in this paper significantly limits enthusiasm for this work.

1) While the authors mention titering the viruses in the methods section, I don't see where that is used in the manuscript. It is thus very hard to understand (for example) if the differences observed in Figure 2C for T cells is due to an improved transduction rate for SRV2, or what could be as little as a 2-fold difference in viral titers in the experiment (which is typically well within stochastic experimental variation). The authors should provide (a) the viral titers upon production of the vector as measured via p24 and/or viral genomes, (b) the MOI for the infection assay, and (c) ideally, transductions conducted at multiple MOI to show exactly how different the SRV2 and RD114 viruses are from each other.

→ Thank you for raising this important concern. (a) The viral genome–based physical titers for each pseudovirus are presented in Fig. 1d. These data show that SRV2 RV consistently yields higher particle numbers upon production compared with RD114 RV. (b) Because the primary aim of this study is to compare the intrinsic transduction efficiency between SRV2 RV and RD114 RV, we determined that comparing at matched MOI would not be appropriate. When vectors differ in physical particle numbers, infectivity, and packaging efficiency, equal-MOI comparisons can introduce misleading interpretations. Therefore, all experiments were performed under conditions in which the qRT-PCR based physical particle numbers of the two pseudoviruses were matched. We consider this approach the most accurate way to compare true gene-delivery efficiency. (c) Moreover, we reasoned that comparing SRV2 RV and RD114 RV using multiple MOIs would still not provide a valid basis for evaluating their relative transduction efficiencies, as such an approach does not resolve the intrinsic envelope dependent differences between the two retroviruses. Both the use of physical-titer matched viral preparations for comparing transduction efficiency and the corresponding cell-to-viral particle ratios (based on physical titers) have been fully described in the Methods section.

Although this analysis was not included in the manuscript, as suggested by the reviewer, the effective MOI was calculated post hoc from the percentage of GFP-positive cells using the Poisson formula $m=-\ln(1-P)$. These low effective MOIs are consistent with the high susceptibility of primary immune cells to retroviral entry, and importantly, they do not affect the relative ranking of transduction efficiencies across pseudotypes. Notably, the effective MOI of SRV2 RV was consistently higher than that of RD114 RV under matched physical titers, clearly supporting the superior transduction efficiency of SRV2.

Experiments	group	T	NK	B
RD114 RV & SRV2 RV	RD114 RV	0.35	0.23	0.04
	SRV2 RV	0.49	0.35	0.06
SRV2 RV & VSV-G LV	SRV2 RV	0.41	0.31	0.06
	VSV-G LV	0.81	0.38	0.27

2) The paper would be significantly improved by better quantification and replication everywhere – multiple batches of virus with reported titers (to allow statistics), multiple transduction experiments to show how transduction rates vary.

→ In accordance with the reviewer's suggestion, we performed independent repeated experiments using T, NK, and B cells activated from PBMCs of different donors, all of which displayed highly consistent across all donors tested. In addition, the viral genome–based physical titers from multiple production batches are presented in Fig. 1d, fulfilling the reviewer's request for quantified viral inputs. Statistical analyses incorporating all repeated datasets have been included in the figures, ensuring rigorous quantification and reproducibility throughout the manuscript.

3) Similarly, it would be necessary to show quantified percentages of cells that are transduced (for example, in Figure 2C, G, K) rather than just the histograms to help understand the differences being shown.

→ As suggested, we added density plots in Fig. 2d, 2i, and 2n to directly present the quantified percentages of transduced cells. This addresses the limitations of histogram-only comparisons and

improves clarity in quantitative interpretation.

4) It is curious the authors do not provide VSVG pseudotyped viruses as comparison given they are by far the predominantly used gene transfer technology. To maximize the benefit of the work, the authors should include this comparison at matched p24.

→ As suggested, we added comparisons with VSV-G pseudotyped lentivirus (LV-VSV-G) in Fig. 5 (CAR) and Supplementary Fig. 1 (GFP). Meanwhile, VSV-G pseudotyped retrovirus was excluded because the produced viral titers were below the measurable range required for meaningful comparison. p24-based normalization is not suitable for comparing SRV2 RV with VSV-G pseudotypes. The amount of Gag-derived p24 packaged per particle differs markedly depending on the envelope, and VSV-G particles in particular incorporate Gag at substantially different stoichiometries and generate a distinct proportion of non-infectious particles. As a result, equalizing viral inputs based on p24 would not reflect equal numbers of functional particles and would lead to a biased comparison of gene-delivery efficiencies. For this reason, we used viral genome–based physical titers, which provide an envelope-independent measure of particle number and therefore offer a more accurate and biologically valid basis for comparing SRV2 RV with VSV-G.

5) The fact that only SRV2 works, and it only works on retroviruses rather than lentiviruses, are both interesting – can the authors provide more hypotheses for why this may be the case?

→ Thank you for this insightful point. To address it, we performed additional experiments using diverse tissue-derived cell types to evaluate whether SRV serotypes exhibit tissue-specific differences. \Although no clear tissue specificity was observed, SRV2 consistently showed the highest infectivity among all tested serotypes. This suggests that amino-acid differences in envelope proteins may underlie differences in viral-entry efficiency. Regarding lentiviral incompatibility, previous literature indicates that the SRV2 cytoplasmic domain (CT) is not efficiently cleaved by the lentiviral protease. We attempted multiple CT modifications but could not achieve titers comparable to VSV-G pseudotyping. Together with findings from Thomas et al. (2019), our data indicate that limited MA–CT interaction in lentiviral systems fundamentally restricts SRV2 pseudotyping. These mechanistic interpretations have been incorporated into the Discussion.

6) The optimizations in Figure 5 would likely be ok in a supplement.

→ As recommended, the optimization experiments originally shown in Fig. 5 have been moved to Supplementary Fig. 3.

7) For the functional data in figure 6, can the authors (a) show the E:T ratio for the experiment, (b) explain why there is such a viability loss in NALM6 when cocultured with untransduced T cells, and (c) explain the seeming CAR-dependent loss in cell viability for HEK cells (which are CD19-)?

→(a) The E:T ratios used in each experiment are now clearly stated in the figure legends. (b) Untransduced T and NK cells possess intrinsic cytotoxic activity, which can contribute to basal lytic effects on target cells such as NALM-6. (c) HEK293T cells are CD19-negative; therefore, CAR-dependent killing does not occur. The observed viability reduction is attributable to innate cytotoxicity of T/NK cells in co-culture. CAR-dependent killing of HEK293T cells was not detected in any condition.

8) Figure 6B – can the authors speculate what is driving higher CAR expression, given the transfer vector is the same? Perhaps perform a VCN assay? The most likely explanation seems like it may be that some of the cells may be multiply transduced, which itself could be affecting function of the CAR-T cells (which is a second order effect of the pseudotyping strategy).

→ As suggested, we performed a ddPCR-based VCN assay(Fig. 5d, supplementary fig. 5c). SRV2 RV derived CAR-T/NK cells exhibited 1.0–3.5 copies per cell, suggesting that some cells may have undergone multiple transduction events, consistent with the reviewer’s hypothesis. However, this VCN range is within the levels commonly reported for clinical-grade CAR-T/NK products and is not associated with tonic signaling or aberrant CAR expression. Recent literature (Chrystel Marton et al., 2025; J. Ma, 2024) also reports that excessively low VCN may compromise therapeutic efficacy,

indicating that the VCN values observed here represent an appropriate range.

9) Figure 6D – I worry that many of these changes are minor enough that they would not be functionally relevant even if they end up statistically significant.

→ We agree with the reviewer that cytokine release and cytolytic activity do not always correlate and may represent mechanistically distinct functional outputs of CAR-T cells. In the initial single-donor experiment, the two CAR-T cells showed a modest difference in cytokine release without an accompanying difference in target-cell lysis, which raised concerns regarding the functional relevance of the cytokine changes.

To rigorously address this point, we repeated the experiments using T cells derived from multiple independent donors. Across these biological replicates, we consistently observed a clear difference in cytotoxic activity between the two CAR-T cells, indicating that the killing potency represents a more stable and donor-independent functional readout. In contrast, cytokine release exhibited greater donor-to-donor variability, resulting in less pronounced differences in the repeated experiments. This variability is consistent with known heterogeneity in T-cell activation states, differentiation subsets, and CAR expression levels across donors.

Importantly, although cytokine levels alone did not reliably predict cytotoxicity, differences in cytokine production observed in the initial experiment may still reflect altered signaling strength that contributes to the functional divergence in killing activity, as supported by the multi-donor cytotoxicity data and differences in CAR expression levels. Collectively, the expanded dataset resolves the reviewer's concern by demonstrating that the two CAR-T cells do differ functionally, with cytotoxic activity serving as the most robust and biologically relevant indicator.

10) Functional data in general would be more convincing if shown in at least 1-2 more donors.

→ As recommended, we performed repeated experiments using T/NK cells activated from additional PBMC donors, and consistent results were observed across all donors.

11) Figure 7- can the authors explain a bit more what the difference is between RV and RV2? Comparing RD114 and RV2-SRV2 feels like an unfair comparison given how much effect the change in transfer vector is clearly having.

→ In the revised manuscript, all retroviral vectors were produced using the same RV2 transfer plasmid backbone. Both RD114 RV and SRV2 RV utilized the identical RV2 backbone, eliminating concerns about unfair comparison due to differences in transfer vectors.

12) Figure 8 – this demonstrates that the CAR is functional, but it completely drops the comparison to any other approaches, limiting its demonstration of utility compared to RD114-Retro, VSV-retro, or (the by far most commonly used) VSV-lenti.

→ Following the reviewer's comment, we added an in vivo comparison between RD114 RV and SRV2 RV derived CAR-T cells (Fig. 6f–j). We also included in vitro comparisons involving VSV-G LV versus SRV2 RV (Fig. 5a–f) and GFP-based transduction analyses (Supplementary Fig. 1), addressing this concern.

Reviewer #3 (Remarks to the Author):

The manuscript by Jeun and colleagues explored a glycoprotein derived from simian retrovirus 2 for pseudotyping of retroviruses. Initially the authors established that of the eight serotypes of SRV the glycoprotein of serotype 2 seems to be the most potent one for transduction of T- and NK cells. Next, the authors produced respective pseudotyped retroviruses. However, production of pseudotyped lentiviral vectors was not feasible. After optimizing the protocol for production of SRV2 pseudotyped retroviral vectors the authors produced vectors encoding the CD19 CAR and demonstrate that this

is advantageous compared to RD114 pseudotyped retroviruses. As a final experiment, the authors show antitumor effects in vivo after administration of the produced CAR-T cells using the SRV2 pseuotyped vector.
This is an interesting study. However, I am not sure whether the advancement in the field is big enough to argue publication in this journal. Moreover, some important information is lacking as specified in the comments below.

Major comments:

• The authors state that the SRV2 RV exhibited approximately 20% more CAR-positive T cells and 7% more CAR-positive NK cells compared to RD114 pseudotyped vectors, respectively. In general, I am not entirely sure whether this advancement especially in NK cells is big enough. In contrast, the data for the modified transfer vector RV2 seem very promising in NK cells. Maybe I missed that information but I think the authors should specify for figure 7B what exactly the modified transfer vector (RV2) looks like. This information should be added to the materials and methods or results section and the phenomenon should be discussed.

→ Thank you for this important comment. In the revised manuscript, all retroviral vectors were generated using the RV2 transfer plasmid backbone, including both RD114 RV and SRV2 RV. Thus, the comparisons presented reflect differences arising from the envelope glycoproteins rather than vector-backbone differences. Although the increase in NK-cell transduction efficiency appears smaller relative to T cells, repeated experiments across multiple donors demonstrated that SRV2 RV consistently showed higher gene-transfer efficiency than RD114 RV in all donors and all cell types.

• In the discussion section the authors claim the results of this manuscript show that SRV-2 pseudotyped retroviruses outperform RD114 pseudotyped retroviruses both in vitro and in vivo. However, there are no results displayed for the RD114 virus in vivo in Figure 8. The authors should perform these experiments.

→ We agree with the reviewer's comment. In Response, we performed an additional in vivo experiment directly comparing RD114 RV and SRV2 RV derived CAR-T cells (Fig. 6f–j). The results show that SRV2 RV derived CAR-T cells exhibit superior tumor-suppression capacity and long-term survival benefit, thereby strengthening the overall conclusion.

• Is it possible to also test the CAR-NK cells in vivo? Have the authors performed these experiments?

→ Evaluating CAR-NK cells in vivo is indeed highly meaningful. However, CAR-NK engraftment, persistence, and donor variability make in vivo NK models technically challenging. We conducted preliminary experiments to explore this possibility, but under the current conditions, reproducible antitumor effects could not be achieved reliably.

Minor comments:

• Figures 2: For subfigures D, H, L it should be stated in the figure legend how many biological and technical replicates were performed? It would be advantageous to add some statistics to these subfigures. The authors display fluorescent intensity for the different groups. How about the percentage of cells, which were transduced?

→ All suggested revisions have been implemented. We performed independent biological replicates using T, NK, and B cells derived from different PBMC donors and included this information in the figure legends. Density plots were added to provide quantified transduced-cell percentages as requested.

• Figure 3: For subfigures C and F, it should be stated in the figure legend how many biological and technical replicates were performed? As already stated in the previous comment the percentage of cells which were transduced should be mentioned (not only the fluorescent intensity)?

→ These points were addressed similarly. Fig. 3b and Fig. 3e were fully revised from fluorescent-

intensity plots to density plot based percentage analyses to provide clearer comparison of transduction efficiencies.

• Whenever suitable in Figures 2 and 3 statistics should be added.

→ Statistical analyses have been incorporated throughout all figures wherever repeated experimental data were available, and significance values are clearly indicated.

※ In addition to the experiments directly requested by the reviewers, we performed several additional studies that we considered essential for strengthening the overall rationale and ensuring the robustness of our conclusions. These supplemental experiments further support the superior performance of SRV2 pseudotyped retroviruses in both viral production and functional CAR-T cell generation.

1. (Fig. 1d)

To more clearly delineate envelope-dependent differences in viral production efficiency, we directly compared the physical titers of RD114 RV and SRV2 RV. This analysis confirmed that SRV2 RV consistently exhibited higher physical titers, indicating enhanced production efficiency.

2. (Fig. 5b)

To evaluate whether SRV2 RV impacts CAR-T cell expansion, we compared the growth kinetics of T cells transduced with RD114 RV, SRV2 RV, and VSV-G LV. All groups showed comparable proliferation, demonstrating that SRV2 RV does not impair CAR-T cell growth. Although not statistically significant, SRV2 RV derived CAR-T cells displayed a modest trend toward higher expansion.

3. (Fig. 5g–j)

To assess whether the enhanced performance of SRV2 RV extends beyond CD19 CAR, we generated FOLR1 CAR and PD-L1 CAR T cells using both RD114 RV and SRV2 RV. In both CAR models, SRV2 RV yielded higher CAR expression and improved target-cell cytotoxicity, further reinforcing the generalizability of SRV2 RV mediated enhancement.

4. (Supplementary Fig. 2b)

To determine whether individual SRV serotypes exhibit tissue-specific tropism, we performed a systematic comparison across cell lines representing distinct tissue origins; A549 (lung), HCT-8 (intestine), HeLa (cervix), and HepG2 (liver). Each serotype-pseudotyped retrovirus was used to deliver GFP into these models to evaluate whether any serotype preferentially transduced a particular tissue type. Across all tissue-derived cell types, SRV2-pseudotyped retrovirus consistently demonstrated the highest transduction efficiency, indicating that its superior performance is not tissue-restricted but likely attributable to intrinsic sequence features of the SRV2 envelope glycoprotein.

REVIEWERS' COMMENTS

Reviewer #1 (Remarks to the Author):

The authors have made extensive and thoughtful revisions to address the majority of concerns raised in the initial review, and the revised manuscript is now substantially improved. This work now provides a well-supported evaluation of SRV2-pseudotyped retroviral vectors for CAR-T/NK engineering, with clear comparative data against the gold-standard RD114 envelope. My further suggestions are as follows:

1.The authors has added VCN data to link transgene copy number with CAR expression and cytotoxicity, a key response to initial requests for mechanistic validation of functional improvements. However, a critical translational limitation remains unaddressed: the study does not examine integration-site preferences or potential genotoxic risk of SRV2-pseudotyped retroviruses. Retroviral insertion near proto-oncogenes is a well-documented cause of leukemogenesis in gene therapy trials (PMID: 37175441), and safety-efficacy balance is central to the clinical potential of this vector system. While comprehensive integration profiling may be beyond the present scope, this limitation must be explicitly acknowledged and discussed in detail in the Discussion section, with a clear note on how this gap impacts the translational interpretability of the study's findings.

- ➔ Thank you for this important comment. We addressed this point by explicitly acknowledging and discussing this limitation in the limitation paragraph of the Discussion section, as suggested.

2.The addition of VCN data raises a critical new interpretative point that the authors must address explicitly: the functional advantage of SRV2-pseudotyped vectors appears to be quantitative rather than qualitative. That is, improved efficacy stems from increased transgene copies per cell, not a unique biological property of SRV2 itself. The authors must explicitly clarify and discuss this distinction in the manuscript to avoid overstating mechanistic novelty. For context, Norberg et al. (PMID:41445185) quantified this exact VCN-dependent effect on CAR-T function, and this reference should be integrated to frame the study's findings appropriately.

- ➔ Thank you for this insightful comment. We addressed this point by explicitly clarifying and discussing this distinction in the paragraph interpreting Figures 1–2 in the Discussion section, and we incorporated the suggested reference to appropriately frame our findings.

3.Mechanism experiments still lack effective supplementation. However, I think the author's reply is reasonable.

4.Extension of the in vivo follow-up substantially strengthens the study. Nonetheless, the relatively small cohort size should be more explicitly acknowledged as a limitation in the Discussion.

- ➔ As pointed out by the reviewer, we acknowledged this point in the limitation paragraph of the Discussion section.

5.Infection parameters and normalization strategies should be more clearly described in the Methods to facilitate reproducibility. For example, incubation time, incubation temperature, and the volume ratio of viral solution to cell suspension.

- ➔ Thank you for this helpful comment. The incubation time and incubation temperature for infection were already described in Methods section 6, "Viral vector-mediated cell transduction." The volume ratio of viral solution to cell suspension was not fixed and could vary between experiments, because infections were performed based on the physical titer. Instead, we stated the ratio of cells to viral physical particles in Method section 6.

6.Ensure consistent terminology when referring to vector types and pseudotyping strategies throughout the manuscript.

- ➔ In accordance with the reviewer’s suggestion, we revised the terminology throughout the manuscript to ensure consistency and unified the vector names as SRV2 RV, RD114 RV, and VSV-G LV, as defined in the Abstract.

7.Please ensure that sample sizes, statistical tests, and definitions of error bars are consistently reported across all figure legends.

- ➔ As the reviewer noted, we checked all figure legends to ensure that sample sizes, statistical tests, and definitions of error bars were reported consistently, and revised them accordingly.

Reviewer #2 (Remarks to the Author):

While I still think many of the differences observed here are quite subtle, the paper is strengthened and there will be interest in continued expansion of alternative pseudo typing strategies- so I think I is reasonable to publish in this form.

Reviewer #3 (Remarks to the Author):

The authors addressed all raised issues and performed additional experiments to address ramaining points and to add respective statistical analyses. Therefore, acceptance of this manuscript can be recommended.
